# dropEst: pipeline for accurate estimation of molecular counts in droplet-based single-cell RNA-seq experiments

**DOI:** 10.1186/s13059-018-1449-6

**Published:** 2018-06-19

**Authors:** Viktor Petukhov, Jimin Guo, Ninib Baryawno, Nicolas Severe, David T. Scadden, Maria G. Samsonova, Peter V. Kharchenko

**Affiliations:** 1 Department of Applied Mathematics, Peter the Great St. Petersburg Polytechnic University, St. Petersburg, Russia; 2 Department of Biomedical Informatics, Harvard Medical School, Boston, MA USA; 3 Center for Regenerative Medicine, Massachusetts General Hospital, Boston, MA USA; 4 Harvard Stem Cell Institute, Cambridge, MA USA; 5 Department of Stem Cell and Regenerative Biology, Harvard University, Cambridge, MA USA

## Abstract

Recent single-cell RNA-seq protocols based on droplet microfluidics use massively multiplexed barcoding to enable simultaneous measurements of transcriptomes for thousands of individual cells. The increasing complexity of such data creates challenges for subsequent computational processing and troubleshooting of these experiments, with few software options currently available. Here, we describe a flexible pipeline for processing droplet-based transcriptome data that implements barcode corrections, classification of cell quality, and diagnostic information about the droplet libraries. We introduce advanced methods for correcting composition bias and sequencing errors affecting cellular and molecular barcodes to provide more accurate estimates of molecular counts in individual cells.

**Electronic supplementary material:**

The online version of this article (10.1186/s13059-018-1449-6) contains supplementary material, which is available to authorized users.

## Background

RNA-seq protocols have been optimized to enable large-scale transcriptional profiling of individual cells. Such single-cell measurements require both improved molecular techniques as well as effective ways to isolate and process a large number of cells in parallel. While single-cell RNA-seq (scRNA-seq) remains a challenging technique, several solutions are being increasingly applied, most notably techniques based on droplet microfluidics such as inDrop [1], Drop-seq [2], and the 10x Chromium platform. In these approaches, cells are encapsulated in water-based droplets together with barcoded beads and necessary reagents within an oil-based flow. This allows the RNA material extracted from each cell to be contained within the droplet and tagged by a unique cellular barcode (CB) carried on the bead.

InDrop and similar approaches pool material from different cells to prepare the library, and rely on computational analysis to recognize the reads originating from the same cell based on the CB contained in the read sequence. The reads also carry a random barcode—a unique molecular identifier (UMI) [3, 4]—that can be used to discount the redundant contribution of reads originating from the same cDNA molecule as a result of library amplification. As such, the primary aim of the data-processing pipeline, including the one presented here, is to provide accurate estimates of the number of molecules that have been observed for each gene in each measured cell—a molecular count matrix. Accurate estimation of such a matrix is crucial, as it commonly provides the starting point for all downstream analysis, such as cell clustering or tracing of cell trajectories.

Several factors complicate the estimation of this molecular count matrix, well beyond simple parsing of the read sequences. First, the procedure must separate reads originating from droplets containing real cells from contributions of empty droplets which can amplify extracellular background transcripts and significantly outnumber the real cells. Some of the droplets may contain damaged or fragmented cells, which complicates such separation. The procedure must also address problems stemming from sequencing errors, particularly errors within the CB or UMIs which result in misclassification of reads. Similarly, skewed distribution of UMIs can lead to biased estimation of molecular counts. Finally, as droplet-based scRNA-seq protocols are still relatively new, detailed diagnostics and multiple quality control steps are typically needed to ensure high-quality measurements and identify likely sources of problems. Given the current lack of such general processing pipelines for droplet-based scRNA-seq, we have set out to provide an open-source implementation.

## Results

We have developed a high-performance pipeline to perform initial pre-processing and analysis of droplet-based scRNA-seq data. The pipeline characterizes the quality of a library using a wide range of diagnostic indicators, filters out artefactual cellular barcodes, evaluates and corrects for potentially confounding effects of uneven UMI coverage, and corrects for UMI and cellular barcode sequencing errors based on molecular similarity measures that do not require prior knowledge of the possible barcode sequences. It is designed to be used with different alignment methods and provides configuration options to accommodate alternative scRNA-seq protocol designs.

### Uneven UMI frequency distribution distorts molecular count estimation

**Fig. 1:**
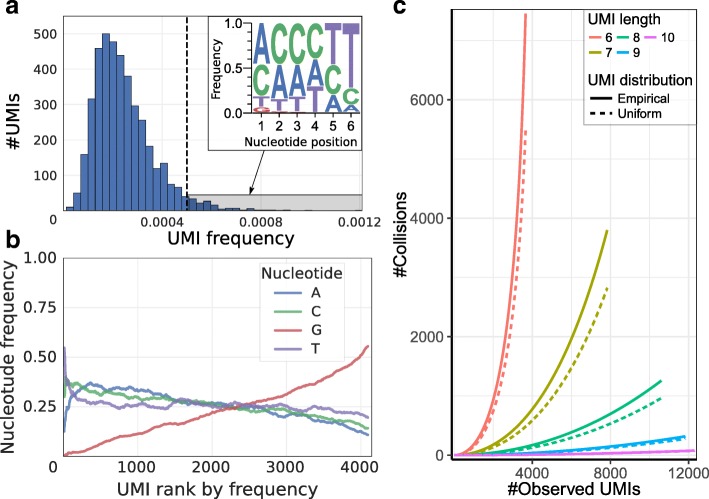
Skewed distribution of UMIs leads to increased number of UMI collisions. **a** Distribution of UMI occurrence frequencies across all genes is shown for mouse embryonic stem (ES) cells (dataset 1). The *top-right inset* shows position-specific nucleotide frequencies of the outlier UMIs (highlighted by *gray shading* on the main plot). Significant skewness of the UMI distribution decreases the effective pool of UMIs. **b** Proportions of different nucleotides in the UMI sequences are shown as a function of the overall UMI frequencies (x-axis orders UMIs so that most frequently occurring UMI sequences have low rank) for the mouse ES cells (dataset 1). **с** Estimated number of UMI collisions as a function of the true gene expression level (x-axis) is shown for different UMI lengths (simulated by trimming 10-nucleotide UMIs; see text). The estimates based on the uniform and empirical UMI distributions are shown. The 10x Chromium human post-transplant BMMC dataset (dataset 7) was used. For short UMIs, the number of collisions observed at highly expressed genes can be comparable to the true number of molecules. Longer UMIs decrease the number of collisions

In UMI-based protocols, the expression magnitude is typically estimated as a number of unique UMIs associated with a given gene in a given cell. If the space of possible UMI sequences is limited, it becomes possible for two separate molecules of the same transcript to be labeled by the same UMI. To account for such UMI collisions, Fu et al. originally proposed a correction based on assumption of uniform distribution of UMIs in the overall dataset [3]. Such correction is rarely used, given relatively large numbers of possible UMIs. Examining droplet data from different protocols, however, we find that UMI frequency distribution tends to be highly skewed, with a small fraction of UMIs contributing to a disproportionately large number of molecules (Fig. 1a, b, Additional file 1: Figure S1). The outlier UMIs with the highest frequencies show lower diversity of nucleotides (Fig. 1a, Additional file 1: Figure S2A). Such biases may arise due to errors generated during the library construction protocol or truncated barcode constructs. Even when such erroneous UMIs are filtered out, the overall UMI distribution remains significantly skewed (Additional file 1: Figure S1B,F), suggesting that a more advanced approach is needed to correct for the impact of UMI collisions. In implementing corrections for the UMI collisions, we therefore moved away from the assumption of a uniform UMI distribution and modeled the true UMI frequency distribution (see “Methods”). This approach is effective at correcting UMI collisions on simulated data (Additional file 1: Figure S2B) and, as we will demonstrate in the next section, provides notable improvements on real data.

### Errors in UMI sequence lead to overestimation of molecular counts

An error introduced into a UMI sequence during the library preparation can be mistakenly interpreted as an additional molecule. Computational corrections have been proposed to avoid such overestimation. The simplest such approach [5] omits for a given gene all UMIs that have an adjacent UMI sequence (Hamming distance equal to 1) with a larger number of reads (as in [6] we refer to this method as *cluster*). Indeed, the probability of two molecules of the same transcript in the same cell being labeled by UMIs of Hamming distance 1 is low, given sufficient size of the UMI pool relative to the number of molecules of that transcript (see “Methods”). However, for moderately expressed genes the observed number of such events exceeds the expected frequency by a factor of ~ 40 (Additional file 1: Figure S3), suggesting that most adjacent UMI occurrences are erroneous. A more complex, network-based solution [6] (referred to here as *directional*) considers a UMI to be erroneous if it has an adjacent UMI with more than twice the number of reads.

An alternative approach, implemented in the 10x Chromium Cell Ranger pipeline [7], uses UMI base call quality to distinguish erroneous UMIs. Examining different droplet-based datasets, we find that the fraction of UMI errors that can be distinguished by lower base call quality varies between datasets, within the range of 29.4–85.6% (Additional file 1: Figure S4). This suggests that a substantial fraction of UMI errors may originate during PCR amplification or other library preparation steps preceding the sequencing itself. Base call quality would not be informative in such cases. Furthermore, the existing methods do not consider the total number of molecules for a given gene, even though the probability of observing adjacent UMIs by chance increases. Such increase is further exacerbated by an uneven distribution of UMI frequencies described in the previous section. For instance, for the inDrop Bone Marrow dataset (dataset 11; see “Methods”), the probability of observing adjacent UMIs under the empirically observed distribution is up to 20% higher than under the uniform distribution (Additional file 1: Figure S5A).

To improve the accuracy of UMI filtering, we developed a Bayesian approach to estimate the posterior probability of a UMI being erroneous based on the gene expression magnitude, the observed number of adjacent UMI sequences, the prior distribution of UMIs, as well as the base-call quality in the position of the nucleotide substitution (see “Methods”). To evaluate performance of different UMI correction approaches, we used artificially trimmed UMIs, where the expected ground truth would be known with high certainty. Specifically, we used the 10x post-transplant bone marrow mononuclear cell (BMMC) data (dataset 7), which has relatively long 10 bp UMIs [8]. Given the lower rate of accidentally observing UMIs at Hamming distance 1 in these longer UMIs, we applied the *cluster* UMI filtering procedure to obtain benchmark ground truth expression estimates for the dataset. We then simulated datasets with shorter UMIs by trimming the UMI sequences, comparing the resulting molecular count estimates to the corresponding full-length benchmark values (see “Methods”). As nucleotide diversity can vary depending on the position in the UMI, we used two versions of trimming: from the front of the UMI sequence and from the back. Both scenarios showed significant excess of UMI collisions compared to what is expected from the uniform distribution (Additional file 1: Figure S5B). More collisions were observed under the front trimming scenario leading to more collisions than with back trimming, indicating lower sequence diversity towards the end of the UMI.

**Fig. 2:**
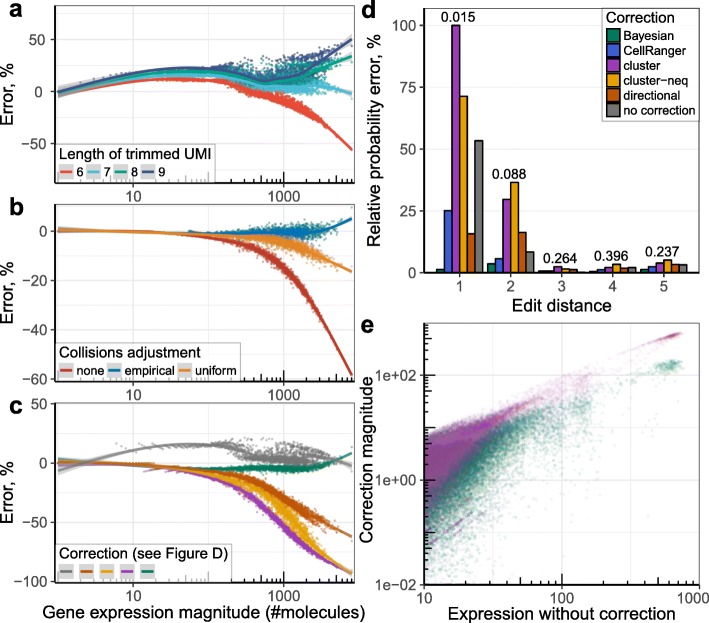
Comparison of UMI collision and sequencing error correction methods. Comparison of UMI collision adjustment and UMI correction algorithms is shown using the 10x post-transplant BMMC dataset (dataset 7). **a** The scatter plot shows percentage error (y-axis) in estimation of the molecular counts for different genes using computationally trimmed UMIs (down to 6–9-nucleotide lengths, as designated by color) from their original 10-nucleotide length, as a function of the full-length UMI estimate (x-axis; see “Methods”). The *line* shows spline-smoothed dependency with the 95% confidence band. *Points* show median y value for a given x. The errors result from two opposing trends, with UMI sequencing errors inflating the resulting count estimates, and UMI collisions deflating the estimates. Shortened UMIs result in a larger number of collisions. **b** The effect of different UMI collision corrections is shown on the 6-nucleotide trimmed UMIs. **c** Comparison of different UMI sequence error correction methods is shown for the 8-nucleotide trimmed UMIs. UMI collisions were corrected using an *empirical* approach in all cases except for “no correction”. **d** We estimated theoretical distribution of edit distances (x-axis) between two randomly sampled UMIs. The theoretical probability of observing a given edit distance is shown as a number above each edit distance group. The histograms show relative absolute difference between this theoretical distribution and observed distributions after the different UMI correction algorithms. For each method and edit distance, the y-axis shows the absolute difference between the observed and theoretical distribution, expressed as a fraction of the theoretical probability of observing that edit distance. **e** Dependency of the magnitude of UMI correction (y-axis) on the expression magnitude without correction (x-axis) is shown. Each point represents a single gene within a cell, pulled across all cells. Genes with expression magnitude < 10 were omitted

While errors in the UMI sequences lead to over-estimation of the molecular abundance, UMI collisions lead to underestimation. The probability of such collisions increases for shorter UMIs, which results in pronounced underestimation of molecular counts at short UMIs (Fig. 2a, Additional file 1: Figure S6). Conversely, overestimation due to sequencing errors is more apparent at longer UMIs. Comparing different UMI collision correction methods, we find that the proposed approach based on the modeling of the empirical UMI frequency distribution shows much better performance than correction based on the uniform UMI distribution assumption (Fig. 2b). We then compared different methods for correcting UMI sequence errors (Additional file 1: Figure S7). In addition to the standard *cluster* algorithm [5], we also evaluated a variant that disallows merging of UMIs of equal sizes (*cluster-neq*). We found that the Bayesian approach proposed here significantly outperforms existing methods (Fig. 2c, Additional file 1: Figure S8). The impact of both collision and sequencing error corrections is most notable for genes within the high expression range, and for datasets with short UMIs (Fig. 2b, Additional file 1: Figure S8). Therefore, for datasets with moderate sequencing depth and long UMIs, analysis can use *cluster* or *directional* algorithms, which are also implemented in the developed pipeline, to reduce computational time.

To further compare the accuracy of UMI corrections introduced by different methods, we examined the distribution of edit distances between two random UMIs following different corrections, comparing it with the expected analytical estimate of the edit distance distribution. Such validation was originally proposed by Smith et al. [6] in describing the *directional* method. Figure 2d shows that, in contrast to other correction methods, the distribution of edit distances after the Bayesian correction closely resembles the theoretical estimation. The differences in probabilities are most notable for edit distances of 1 and 2.

### Correction of the cellular barcode sequence errors

**Fig. 3:**
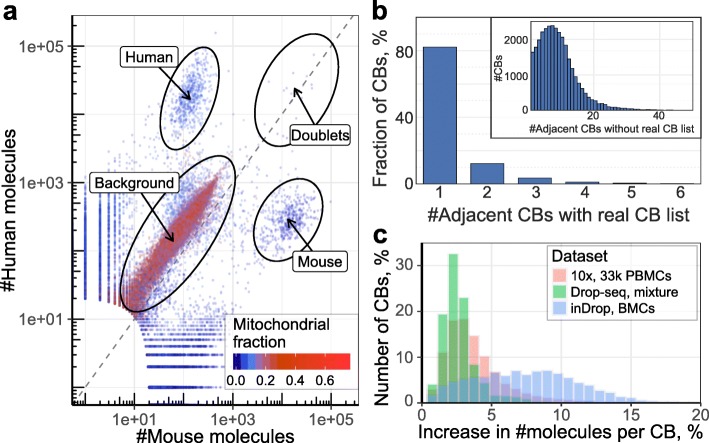
Correcting for cellular barcode errors. **a** The number of molecules mapping to human and mouse genomes in a human–mouse Drop-seq dataset (dataset 12) is shown for each cell (*points*) on a log scale. The plot shows annotations of high-confidence cells for each organism, doublets, and background barcodes. **b** The number of equidistant adjacent CBs of larger size (i.e., number of molecules) is shown for each of the observed CBs in the mouse embryonic stem cell dataset (dataset 1). The main plot shows adjacent CBs selected from an a priori known set of valid CB sequences. The *inset* shows counts of adjacent CBs selected from all CB sequences observed in the dataset. **c** To illustrate the effect of CB corrections, the plot shows the increase in number of molecules per CB (x-axis) following a CB merge correction procedure, relative to the original size. The 10× 8k PBMC (dataset 13), Drop-seq human–mouse mixture (dataset 12), and inDrop BMC (dataset 11) datasets are shown

The number of different cellular barcodes (CBs) in a droplet-based library normally exceeds the number of actual encapsulated cells by several fold (Additional file 1: Figure S9). Similar to issues encountered for UMIs, additional CBs can result from sequence errors introduced during library construction or sequencing. This would result in material from one droplet being mistakenly split up into several different CBs. Alternatively, additional CBs may also be empty droplets that did not encapsulate a real cell, but instead captured background RNA or cell debris together with an indexing bead [9]. To evaluate whether this is a significant factor, we examined the read composition in published 10x [10] and Drop-seq [2] datasets (datasets 4 and 12) with mixtures of human and mouse cells (see “Methods”). We found that in these experiments, background barcodes contained a constant ratio of mouse and human reads, consistent with the idea of extracellular background admixture (Fig. 3a, Additional file 1: Figure S10). However, the absolute abundance was dependent on the total size of each barcode, suggesting more complex interaction with library preparation and processing. Furthermore, we were not able to reconstruct a uniform background “transcriptome”, as the identity of the admixed transcripts varied considerably among different barcodes.

We first examined methods for correcting CB sequence errors. In doing so we considered two scenarios: one where a list of possible valid CB sequences is known (e.g., 10x or inDrop), and another where CBs can be an arbitrary nucleotide sequence (e.g., Drop-seq). Pre-designed CB sequences are typically evenly spaced in the sequence space, and replacing an erroneous CB with the closest matching valid CB sequence is an effective strategy. The space of potential valid CBs can be further narrowed down by taking into account that the valid CB shouldn’t have fewer counts than the erroneous CB. However, if the list of possible valid CBs is unknown, or if there are many similar CBs (e.g., short barcodes), the number of possible merge targets increases significantly (Fig. 3b). To accurately determine the probability that two CBs originated from one CB, we used UMI–gene composition similarity, which evaluates the likelihood that two independent cells will end up producing equivalent UMI–gene combinations (see “Methods”). This method was compared with the simpler approach, which, for every CB, checks if another CB exists with similar CB sequence (Hamming distance ≤ 2) and containing more molecules, and then merges such CBs.

To compare the two approaches, we again examined the 10x [10] and Drop-seq [2] human/mouse mixture datasets (datasets 4 and 12). As the list of real barcodes is known for the 10x data, we compared the merges introduced by the two approaches with the list of real barcodes. The proposed molecular content-based merge algorithm outperforms the simple approach (Table 1) and shows performance similar to the scenario when the list of true barcodes is known. The proposed approach also reduces the fraction of CBs erroneously merged across the two organisms to negligible levels, while such a fraction is notable with the original method. The differences become more pronounced for larger datasets. For instance, analyzing the 10x 33k peripheral blood mononuclear cell (PBMC) data (dataset 10) [11], 10x Cell Ranger identifies 33,148 real cells, making use of the known barcode list. Reanalysis using the proposed merge procedure (without the knowledge of true barcodes) identifies 31,164 cells containing at least 100 genes. By comparison, the simple approach over-merges, yielding only 12,388 cells at the same minimal gene number threshold. Despite the low false-positive merge rate, the proposed approach can increase the number of molecules per cell (up to 15%; see Fig. 3c).

**Table 1:** Analysis of merge targets on human–mouse mixture datasets

Dataset	Merge type	Number of merges	Fraction of mixed merges	Similarity to merge with barcodes
10x	Poisson	8999	0.58%	99.74%
10x	Known barcodes	8985	0.62%	100%
10x	Simple	21,827	32.96%	20.67%
Drop-seq	Poisson	15,186	0.83%	–
Drop-seq	Simple	26,154	8.74%	–

### Recognizing damaged or low quality cells

The number of molecules associated with a given CB generally provides reasonable criteria for selecting real cells [1, 7]. Similarly, CBs with very few associated reads likely represent empty droplets. However, classifying CBs in the intermediate range poses a challenge. The intermediate size CBs likely contain damaged or dying cells from which relatively little mRNA material could be recovered [9]. This complicates the optimization of a size separation threshold. Such low-quality cells could also cover a range of sizes, making the use of a single size cutoff ineffective.

Classification of low quality cells was examined by Ilicic et al. [9], where a support vector machine (SVM) classifier was trained based on examination of cell morphology from microscopy data prior to lysis and library preparation. As such data are difficult to obtain for droplet-based techniques, and an existing SVM cannot be directly applied to different protocols or cell types, we aimed to develop a self-contained approach that would not require high-quality experiments for training. While the true labels for low- and high-quality cells are not available, we argued that large cells initially include a large fraction of high-quality cells and small cells include a very low fraction of high-quality cells. We then aimed to train a classifier to distinguish high-quality cells based on a limited set of technical features (see “Methods”), taking into account that the initial labels of the training set will contain some fraction of errors. The tolerance of different classifiers to training set errors can vary considerably. We evaluated performance of several appropriate approaches (KDE [12], Random Forest [13], and Robust Gaussian Processes [14]; see “Methods”). In addition to the cross-validation score, we measured robustness of the classifiers with respect to: removal of a random 20% of the training data (fivefold cross-validation; Table 2); introduction of artificial noise into the data (Additional file 1: Figure S11A, B); and narrowing/widening of the thresholds used to separate large and small cells for the initial label assignment (Additional file 1: Figure S11C). Based on the resulting performance and runtime complexity (e.g., Robust Gaussian Processes has a high complexity of O(n^3^) relative to the number of samples) we chose the Kernel Density Estimation (KDE) classifier.

**Table 2:** Fivefold CV comparison of classifiers

Classifier	Sensitivity on CV (%)	Specificity on CV (%)	Stability on class 1 (%)	Stability on class 0 (%)
KDE	90.4 (±0.8)	91.1 (±3.4)	89.8 (±2.9)	97.3 (±1.3)
Random Forest	89.6 (±2.3)	92.7 (±1.6)	87.3 (±7.3)	97.7 (±1.3)
Robust GP	87.7 (±2)	94.8 (±2.2)	85.3 (±0)	99.3 (±0.5)

Mean ± standard deviation values are shown**.** Here, class 1 is high-quality cells, class 0 is low-quality cells

**Fig. 4:**
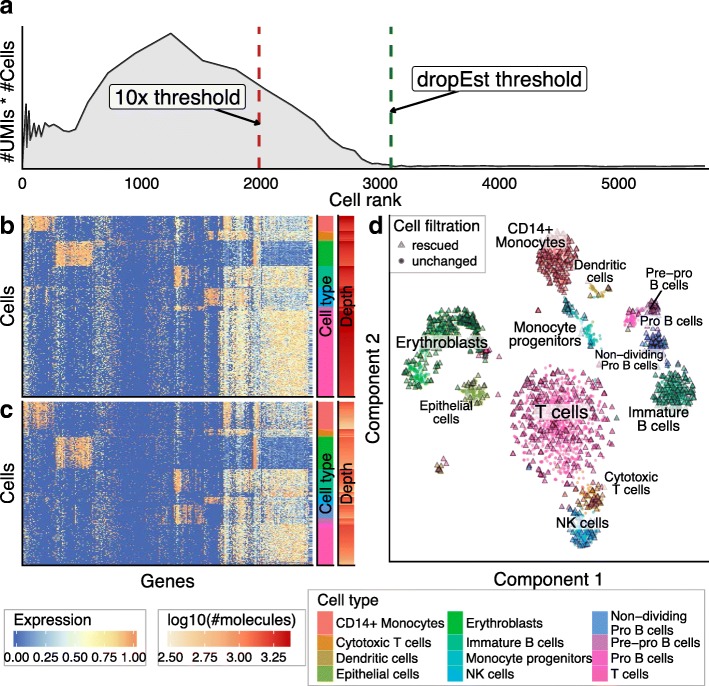
Selection of the optimal size threshold for the 10x BMMC dataset. These plots show comparison of dropEst and 10× Cell Ranger strategies for initial selection of number of real cells in the 10x BMMC dataset (dataset 8). **a** The distribution of molecular mass across CBs of different sizes. The y-axis shows the number of UMIs per cell multiplied by the number of cells with a similar number of UMIs. The cells are ranked by their size (number of UMIs), with the largest cells positioned near 0 (see “Methods”). Such “molecular mass” plots can be used to estimate the number of real cells in a dataset. Here, the peak centered around x = 1200 represents real cells. The *vertical dashed lines* show size-based thresholds, as determined by Cell Ranger (*red*) and dropEst (*green*). dropEst threshold admits 1105 additional cells. **b** The heatmap shows gene expression profiles of cluster-specific genes for the cells that were admitted by both 10x and dropEst thresholds. Expression levels of different genes (columns) are shown by color. Cells (rows) are grouped by cluster (see *cluster bar* on the *right*), and then ordered descending by number of molecules (the *depth bar* on the *right*). Genes (rows) were clustered using hierarchical clustering. See “Methods” for details. **c** Similar to **b**, the heatmap shows expression of the same genes in the set of an additional 1105 cells admitted by the dropEst threshold procedure. The additional cells show expression patterns consistent with their assigned clusters. **d** t-SNE visualization of the 10x BMMC dataset. All cells which pass both Cell Ranger and dropEst thresholds are shown as *circles*. Cells which were admitted only with the dropEst threshold are shown as *triangles*

Cell size-based thresholding approaches, such as the one implemented by the Cell Ranger software, can provide a reasonable guess for the initial separation of high-quality cells. We implemented a modified threshold-selection method that does not require assumptions about the number of true cells. For most datasets, the determined thresholds are similar to those chosen by the Cell Ranger approach; however, the difference was notable for some datasets. For instance, for the 10x human BMMC dataset (dataset 8), the threshold determined by our approach recovers 1105 additional cells that show subpopulation-specific expression signatures (Fig. 4, Additional file 2: Table S4). We note that these additional cells are not evenly distributed across different subpopulations but preferentially augment certain subpopulations, such as the non-dividing subgroup of pre-B cells (expressing both IGLL5 and CD37). The cells in these populations show smaller average library sizes (number of detected molecules), explaining their over-representation within the tail of the cell size distribution. For details on cell type annotation see Additional file 1: Figures S12–S14 and Additional file 2: Tables S1–S3.

**Fig. 5:**
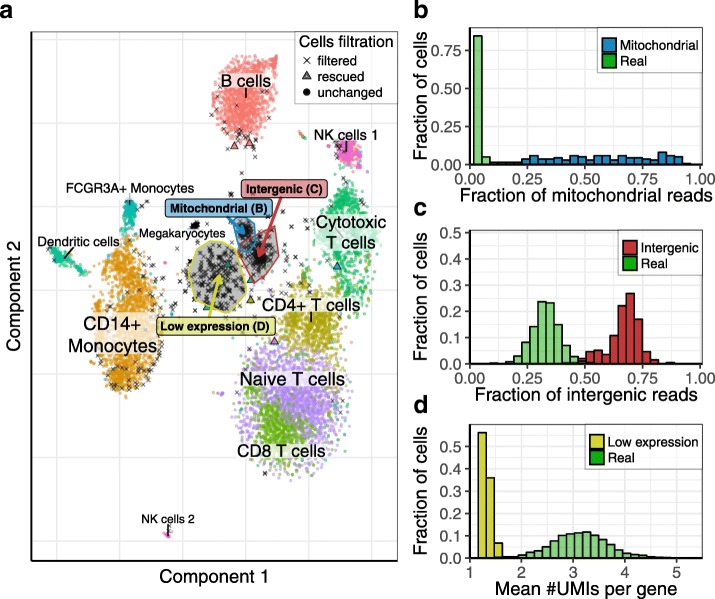
Filtration of low-quality cells for the 10× 8k PBMC dataset. This figure shows the result of the KDE-based algorithm for the filtration of low-quality cells on the 10× 8k PBMC dataset (dataset 13). **a** t-SNE visualization of the cell subpopulations; only cells which either passed the size threshold or have a quality score > 0.9 are shown. Cells passing the dropEst size threshold and having a quality score ≥ 0.1 are shown with *circles*. A few cells falling below the size threshold but with a high (> 0.9) quality score are shown with *triangles*. Cells passing the size threshold but with a low (< 0.1) quality score are considered as filtered and are shown with *black crosses*. Most filtered cells originated form three distinct clusters, marked by a high fraction of intergenic or mitochondrial reads and a low number of reads per UMI (see labels). **b**–**d** Distributions of distinguishing characteristics (x-axes) are compared between clusters of low quality cells and the real cell population. Here, we consider a cell to be real if it passes the size threshold and has a quality score > 0.9

**Fig. 6:**
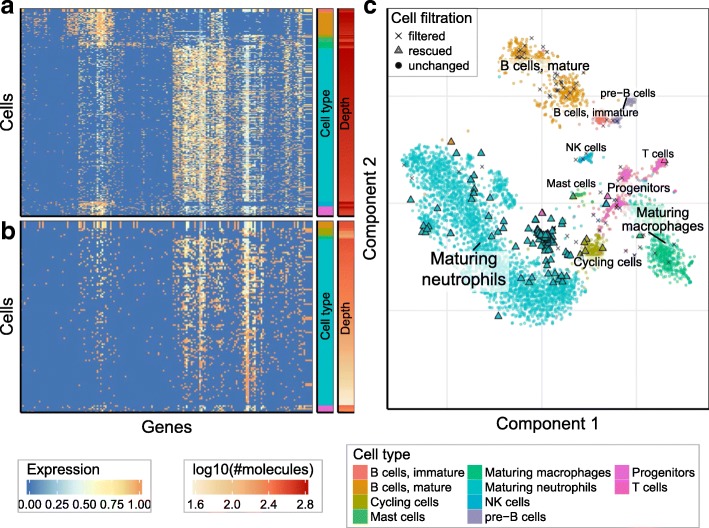
Filtration of low-quality cells for the inDrop mouse BMC dataset. This figure shows the result of the KDE-based algorithm for filtration of low-quality cells in the inDrop mouse BMC dataset (dataset 11). **a**, **b** Similar to Fig. 4b, c, the heatmap shows expression of cluster-specific genes in cells with high quality scores (> 0.9) that were identified above the size-based threshold (**a**), and “rescued” below the size-based threshold (**b**). **c** t-SNE visualization of the dataset, similar to Fig. 5a

KDE-based quality scores refine identification of high-quality cells around size-based thresholds (Additional file 1: Figures S15 and S16). While the quality scores overall show expected correlation with cell size, some of the smaller cells are able to attain high scores, and some of the large cells are assigned low scores (Additional file 1: Figure S15A). For the 10× 8k PBMC dataset (dataset 16), the quality scores pick up an additional 170 cells relative to the size threshold determined by Cell Ranger (Additional file 1: Figure S15B,C, Additional file 2: Table S5). When compared to our own threshold-determination method, the quality scores correctly filter out poor-quality cell clusters (Fig. 5). In the context of inDrop mouse pancreatic cells [15] (Additional file 1: Figure S17, Additional file 2: Table S6) and the inDrop mouse BMC dataset (dataset 11; Fig. 6, Additional file 2: Table S7), quality scores recover additional cells that show expression patterns consistent with the major subpopulations.

## Discussion

Droplet-based microfluidics protocols and other high-throughput methods are enabling production of large single-cell RNA-seq datasets (10^3^–10^6^ cells). Complex barcoding schemes employed by such methods require in-depth computational analysis to achieve accurate recovery of molecules associated with different cells and genes. In order to avoid collisions of cellular barcodes, large numbers of cells necessitate longer CBs, increasing the probability that a sequence error will be introduced into a CB during the bead construction steps [1], library preparation procedures, or library sequencing. We show that for many such errors there are multiple equidistant CBs from which the molecule may have originated. The implemented solution, which merges CBs based on the probabilistic assessment of the molecular overlap between the CBs, provides accurate correction even in cases when the set of possible valid CBs is not known in advance.

Errors affecting molecular barcodes (UMIs) pose a similar challenge, which in this case is driven by increasing sequencing depth of individual cells. This has been recognized by earlier studies [5], and several correction strategies have been proposed. We show that the overall distribution of UMI sequence occurrences is not uniform, and the resulting bias reduces the effective UMI space leading to increased number of UMI collisions in well-expressed genes and deflated molecular counts. Some of the UMI errors appear to result from occurrence of aberrant library molecules incorporating mononucleotide primers, such as poly(T) into the UMI position. On the other hand, point mutations in UMIs and aberrant base calls can lead to inflated molecular counts. While most UMI errors can be mitigated experimentally by increasing the UMI length, we show that taking into account empirical distribution of UMI frequencies allows adjustment for both UMI collision and sequence error effects.

Even with corrections of CB sequence errors, most of the CBs encountered in the current droplet-based datasets do not represent real cells. These additional molecules may originate from empty droplets capturing extracellular background. Indeed, examination of mouse–human dataset mixtures suggests that smaller CBs have a higher cross-organism contamination fraction than one would expect from extracellular background. In addition to empty droplets, some of the low-magnitude CBs may represent damaged, dying, or dead cells, as well as cells that were not successfully measured for other reasons. The challenge of identifying damaged cells has been previously examined by Ilicic et al. [9] in the context of the Fluidigm C1 protocol, where the proportion of low-quality cells is typically in the range of 10–40%. This fraction can be much higher in the inDrop data (e.g., 90% of CBs), and obtaining microscopy-based labeling for the classification is challenging given the rapid flow within the devices. We instead explored application of fault-tolerant classifiers to identify technical features consistent with an imperfect initial separation of high-quality cells based on the size criteria alone. Such an approach is able to pick up relatively large cells that resemble poorly measured cells based on their technical features, and rescue some of the smaller cells that look consistent with the high-quality tail of the cell distribution.

## Conclusions

Overall, we hope that the developed pipeline will facilitate analysis of droplet-based single-cell RNA-seq data, providing helpful diagnostics (see Additional file 3: Supplementary Note 1 for an example of a dropEst pipeline report) and improving the accuracy of the resulting expression estimates.

## Methods

The dropEst pipeline operates in three phases: i) identifier parsing phase; ii) read mapping phase; and iii) filtering and quality control phase. The first phase takes as an input FASTQ files containing paired-end read and index data. The output of this phase is a modified FASTQ file with reads which can be aligned to a transcriptome reference during the second phase using a standard splice-aware aligner (e.g., STAR [16] or TopHat 2.1.0 [17], which was used in our work). The third phase takes BAM files with the aligned reads [18] and a gene annotation file in GTF format. BAM files produced by the 10x Cell Ranger pipeline can also be provided when running this stage. The result of pipeline is an R-readable file that contains molecular count matrix and other processed information, as well as a report with diagnostic information on the library. Sample runtimes for different pipeline steps are shown in Additional file 2: Table S8.

### Correction of UMI collisions

In cases when the number of UMIs per gene is comparable to the total UMIs pool size, the gene expression level will be underestimated [3] and needs to be adjusted by taking UMI collisions into account. Fu et al. [3] assumed uniform distribution of UMI probabilities, and then the number of unique UMIs expected for number of molecules *n* from a UMI pool of size *m* is *k* = *m*[1 − *e*^−(*n*/*m*)^], thus $n=-m\ \mathit{\ln}\left(1-\frac{k}{m}\right)$. To account for a non-uniform UMI distribution observed in the droplet datasets, we estimated *n*(*k*, *m*) by modeling the collisions process. Let’s assume that we have a gene with *k* distinct UMIs *G*_*k*_ and a distribution of UMI probabilities *P*(*u*_*i*_). In this case, the probability of observing a new distinct UMI is $p\left({u}^{\prime}\notin {G}_k\right)={\sum}_{i=1}^mp\left({u}_i\right)\ast {\left(1-p\left({u}_i\right)\right)}^{n(k)}$. The expected number of collisions prior to obtaining a new distinct UMI is equal to *p*(*u'* ∉ *G*_*k*_)^−1^ and *n*(*k* + 1) = *n*(*k*) + *p*(*u'* ∉ *G*_*k*_)^−1^. Thus, we can use a step-by-step procedure to estimate *n*(*k*) ∀ *k* ∈ 1 : *m*.

To validate the developed method, we simulated UMI collisions using a bootstrap procedure (Additional file 1: Figure S2). To do so, we estimated the number of collisions by sampling UMIs from the common distribution one by one, until the expected number of distinct UMIs was reached.

### Correction of UMI sequence errors

To determine whether two UMIs represent technical variations (sequencing errors) of the same UMI, we use a Bayesian approach to estimate the number of errors within each gene within each cell by maximal likelihood. To do so, we can model the process of generating UMI composition.

Given two UMIs within a gene, we considered the following features:

- *U*, sequence of the first UMI.
- *u*, sequence of the second UMI.
- *R*, number of reads for the first UMI.
- *r*, number of reads for the second UMI, *r* ≤ *R*.
- *N*_*S*_, number of adjacent (Hamming distance of 1) UMIs for the UMI *U* with the number of reads *r'* : *r'* ≤ *R*.
- *N*_*L*_, number of adjacent UMIs for the UMI *U* with the number of reads *R'* : *R'* > *R*.
- *S*_*g*_, number of UMIs in the gene.
- *q*, mean Phred quality score of the distinguishing position for the UMI *u*. Here, we use mean value as we already include parameter *r*, which is strongly correlated with the total (sum) quality score.

Let us denote:

- #*Errors* is the number of erroneous UMIs
- #*Real* is the number of real UMIs

We can divide set Ω of all adjacent UMIs into two sets: Ω_*E*_ and Ω_¬*E*_, which means erroneous and real UMIs, respectively. We aim to estimate:

$$ p\left(\# Errors=k\right)={\sum}_{\begin{array}{c}{\Omega}_E:\left|{\Omega}_{\mathrm{E}}\right|=k,\\ {}{\Omega}_{\neg E:}\ \left|{\Omega}_{\neg E}\right|=\left|\Omega \right|-k\end{array}}p\left({\Omega}_E,{\Omega}_{\neg E}\right). $$

The probability of the state with separation Ω_*E*_, Ω_*R*_ within a gene of size *S*_*g*_ is:

$$ p\left({\Omega}_E,{\Omega}_{\neg E}\right)=p\left(R,\overrightarrow{r},\overrightarrow{q},{N}_S=\dim \left(\overrightarrow{r}\right),{N}_L,U,{S}_g, Err\left({\Omega}_E\right),\neg Err\left({\Omega}_{\neg E}\right)\right)= $$

$$ =p\left(\overrightarrow{q}|\overrightarrow{r},R,{N}_S,{N}_L,{S}_g, Err\left({\Omega}_E\right),\neg Err\left({\Omega}_{\neg E}\right)\right)\ast $$

$$ \ast p\left( Err\left({\Omega}_E\right),\neg Err\left({\Omega}_{\neg E}\right)|\overrightarrow{r},R,U,{N}_S,{N}_L,{S}_g\right)\ast p\left(\overrightarrow{r},R,U,{N}_S,{N}_L,{S}_g\right) $$

Here, event *Err*(Ω_*E*_) means that all UMIs from Ω_*E*_ were generated from *U* by an error $\left( Er{r}_{U,{u}^{\prime }}\forall {u}^{\prime}\in {\Omega}_E\right)$. We can omit $p\left(\overrightarrow{r},R,U,{N}_S,{N}_L,{S}_g\right)$ as it doesn’t depend on the separation Ω_*E*_, Ω_¬*E*_. We can also assume independence of properties of Ω_*E*_ and properties of Ω_¬*E*_:

Thus:

$$ p\left({\Omega}_E,{\Omega}_{\neg E}\right)\approx p\left(\overrightarrow{q}|\overrightarrow{r},R,U,{N}_S,{N}_L,{S}_g, Err\left({\Omega}_E\right),\neg Err\left({\Omega}_{\neg E}\right)\right)\ast $$

$$ \ast p\left( Err\left({\Omega}_E\right),\neg Err\left({\Omega}_{\neg E}\right)|\overrightarrow{r},R,U,{N}_S,{N}_L\right)= $$

$$ =p\left(\overrightarrow{q_{\Omega_{\neg E}}}|\overrightarrow{r},R,U,{N}_S,{N}_L,{S}_g,\neg Err\left({\Omega}_{\neg E}\right)\right)\ast p\left(\overrightarrow{q_{\Omega_E}}|\overrightarrow{r},R,U,{N}_S,{N}_L,{S}_g, Err\left({\Omega}_E\right)\right)\ast $$

$$ \ast p\left( Err\left({\Omega}_E\right),\neg Err\left({\Omega}_{\neg E}\right)|\overrightarrow{r},R,U,{N}_S,{N}_L,{S}_g\right) $$

Let us make the following independence assumptions:

$$ p\left({\Omega}_E,{\Omega}_{\neg E}\right)\approx p\left(\overrightarrow{q_{\Omega_{\neg E}}}|\neg Err\left({\Omega}_{\neg E}\right)\right)\ast p\left(\overrightarrow{q_{\Omega_E}}|\  Err\left({\Omega}_E\right)\right)\ast $$

$$ \ast p\left( Err\left({\Omega}_E\right),\neg Err\left({\Omega}_{\neg E}\right)|\overrightarrow{r},R,U,{N}_S,{N}_L,{S}_g\right) $$

Also, we can assume distributions of #*Errors* and #*Real* to be independent. Then, we can write the last part as:

$$ p\left( Err\left({\Omega}_E\right),\neg Err\left({\Omega}_{\neg E}\right)|\overrightarrow{r},R,U,{N}_S,{N}_L,{S}_g\right)= $$

$$ =p\left(\left(\# Errors=\left|{\Omega}_E\right|\right),\left(\# Real=\left|{\Omega}_{\neg E}\right|\right)|\overrightarrow{r},R,U,{N}_S,{N}_L,{S}_g\right)= $$

$$ =p\left(\# Errors=\left|{\Omega}_E\right||\overrightarrow{r},R,U,{N}_S,{N}_L,{S}_g\right)\ast p\left(\# Real=\left|{\Omega}_{\neg E}\right||\overrightarrow{r},R,U,{N}_S,{N}_L,{S}_g\right) $$

Let us make the following additional independence assumptions:

$$ p\left( Err\left({\Omega}_E\right),\neg Err\left({\Omega}_{\neg E}\right)|\overrightarrow{r},R,U,{N}_S,{N}_L,{S}_g\right)= $$

$$ =p\left(\# Errors=\left|{\Omega}_E\right||\overrightarrow{r_{\Omega_E}},R,{N}_S\right)\ast p\left(\# Real=\left|{\Omega}_{\neg E}\right||U,{N}_S,{N}_L,{S}_g\right) $$

As several erroneous UMIs can have the same sequence, we adjust the overall probability:

$$ p\left( Err\left({\Omega}_E\right),\neg Err\left({\Omega}_{\neg E}\right)|\overrightarrow{r},R,U,{N}_S,{N}_L,{S}_g\right)=p\left(\# Real=\left|{\Omega}_{\neg E}\right||U,{N}_S,{N}_L,{S}_g\right)\ast $$

$$ \ast {\sum}_{i=\left|{\Omega}_E\right|}^{N_S}p\left(\left(\# Error{s}_T=i\right),\left(\# Collisions=i-\left|{\Omega}_E\right|\right)|\overrightarrow{r_{\Omega_E}},R,{N}_S\right) $$

where *p*(#*Collisions*) is the probability that the number of erroneous UMIs which have the same sequence is equal to #*Collisions*. #*Errors*_*T*_ is the total number of errors, including those that were not observed. We can assume that :

$$ p\left( Err\left({\Omega}_E\right),\neg Err\left({\Omega}_{\neg E}\right)|\overrightarrow{r},R,U,{N}_S,{N}_L,{S}_g\right)=p\left(\# Real=\left|{\Omega}_{\neg E}\right||U,{N}_S,{N}_L,{S}_g\right)\ast $$

$$ \ast {\sum}_{i=\left|{\Omega}_E\right|}^{N_S}p\left(\# Collisions=i-\left|{\Omega}_E\right||\# Error{s}_T=i\right)\ast p\left(\# Error s=i|\overrightarrow{r_{\Omega_E}},R,{N}_S\right). $$

which yields a complete formula for the overall probability:

$$ p\left({\Omega}_E,{\Omega}_{\neg E}\right)\approx p\left(\overrightarrow{q_{\Omega_{\neg E}}}|\neg Err\left({\Omega}_{\neg E}\right)\right)\ast p\left(\overrightarrow{q_{\Omega_E}}|\  Err\left({\Omega}_E\right)\right)\ast p\left(\# Real=\left|{\Omega}_{\neg E}\right||U,{N}_S,{N}_L,{S}_g\right)\ast $$

$$ \ast {\sum}_{i=\left|{\Omega}_E\right|}^{N_S}p\left(\# Collisions=i-\left|{\Omega}_E\right||\# Error{s}_T=i\right)\ast p\left(\# Error{s}_T=i|\overrightarrow{r_{\Omega_E}},R,{N}_S\right) $$

Direct estimation of the distribution *p*(#*Errors* = *k*) =  ∑ *p*(Ω_*E*_, Ω_¬*E*_) requires an exhaustive search over all subsets of Ω, which takes *O*(2^|Ω|^) operations, making it computationally intractable. To optimize this estimation, let us assume that we can estimate *p*(*u* ∈ Ω_*E*_). Furthermore, we can assume that event (*u* ∈ Ω_*E*_) is equal to (*u′* ∈ Ω_*E*_) ∀ *u′* : *p*(*u′* ∈ Ω_*E*_) ≥ *p*(*u* ∈ Ω_*E*_). The opposite would be true as well: event (*u* ∉ Ω_*E*_) is equal to (*u′* ∉ Ω_*E*_) ∀ *u′* : *p*(*u′* ∈ Ω_*E*_) < *p*(*u* ∈ Ω_*E*_). Thus, we can order all UMIs according to this probability and reduce the search space to *O*(|Ω|). In practice, we don’t even need to estimate *p*(*u* ∈ Ω_*E*_), because for a fixed *U* it depends only on two parameters: *r* and *q*. Moreover, it decreases exponentially with increasing *r* (see explanation below), but there is no such fast dependency for *q*. So, we can order UMIs by descending *r* (first), and then by *q* (second).

### Estimating probabilities

#### Estimation of the quality probabilities

Components of $\overrightarrow{q_{\Omega}}$ can be assumed to be independent. Thus:

$$ p\left(\overrightarrow{q_{\Omega_E}}|\  Err\left({\Omega}_E\right)\right)={\prod}_{u\in {\Omega}_E}p\left(q| Er{r}_{U,u}\right);p\left(\overrightarrow{q_{\Omega_{\neg E}}}|\neg Er r\left({\Omega}_{\neg E}\right)\right)={\prod}_{u\in {\Omega}_{\neg E}}p\left(q|\neg Er{r}_{U,u}\right). $$

Distribution *p*(*q*| ¬*Err*_*U*, *u*_) can be estimated as *p*(*q*| ¬*Err*_*U*, *u*_) ≈ *p*(*q*), since an event ¬*Err*_*U*, *u*_ does not by itself gurantee that *u* is real as *u* can be produced by an error from a UMI other than *U*. Though distribution *p*(*q*) is continuous, we estimated quantized version of this distribution through the following procedure. First, we estimated *k* uniformly distributed quantiles. All quantiles with the difference in indexing variable *q* less than 10^−5^ were assumed to be equal and merged. Then, each value of *q* was rounded off to the nearest quantile. As a result we obtained a discrete distribution with no more than *k* possible values of the indexing variable. In this work we used *k* = 15.

To estimate *p*(*q*| *Err*_*U*, *u*_) we created a training sample, which contained only pairs of UMIs where *u* occurred because of an error in *U*. Such a set was assembled by choosing genes containing two adjacent UMIs only. The theoretical probability *p*(*u*, *U*| *S*_*g*_ = 2, ¬*Err*_(*U*, *u*)_) is negligible. We therefore expect almost all such events to have occurred because of an error in *U*. Such a training sample is representative because *q* is independent of *S*_*g*_. Because values of *q* are discrete following the quantization, estimation of *p*(*q*| *Err*_*U*, *u*_) becomes straightforward.

#### Estimation of the number of real UMIs

Probability *p*(#*Real* = |Ω_¬*E*_|| *U*, *N*_*S*_, *N*_*L*_, *S*_*g*_) depends on the large numbers of parameters, making the training approach impractical. We use theoretical estimation of *p*(#*Real* | *U*, *N*_*L*_, *S*_*g*_) (see the algorithm below), assuming that:

$$ p\left(\# Real=\left|{\Omega}_{\neg E}\right||U,{N}_S,{N}_L,{S}_g\right)=p\left(\# Real=\left|{\Omega}_{\neg E}\right||U,\# Real\le {N}_S,{N}_L,{S}_g\right)= $$

$$ \frac{p\left(\# Real=\left|{\Omega}_{\neg E}\right|\ |\ U,{N}_L,{S}_g\right)}{\sum_{n=0}^{N_S}p\left(\# Real=n\ |\ U,{N}_L,{S}_g\right)}. $$

Let us denote the following notations:

- *L*, length of an UMI.
- *N*_*UMI*_, total number of possible UMIs (in most cases is equal to 4^*L*^).
- *K*, maximum number of the adjacent UMIs (in most cases is equal to 3*L*).
- *p*_*Adjacent*_ = *p*_*Adjacent*_(*U*), probability to observe an UMI, adjacent to *U*. It is equal to $\sum \limits_{u\in Adjacent(U)}p(u)$.
- *N*′, total number of real adjacent UMIs for the UMI *U*.

To estimate the distribution of *p*(#*Real*| *S*_*g*_, *U*, *N*_*L*_) we use the following assumption:

$$ P\left(\# Real\ge n|{S}_g,U,{N}_L\right)=P\left({N}^{\prime}\ge n+{N}_L|{S}_g,U,{N}^{\prime}\ge {N}_L\right)=\frac{P\left({N}^{\prime}\ge n+{N}_L|{S}_g,U\right)}{\Sigma_{k={N}_L}^KP\left({N}^{\prime}\ge k|{S}_g,U\right)}. $$

The distribution *p*(*N*′| *S*_*g*_, *U*) was estimated by modeling the process of picking UMIs from a pool. Suppose that we have already picked *s* UMIs, and we have *k* different adjacent UMIs. Let us denote this state as (*k*, *s*). This state can occur in one of the following situations:

1. We were previously in the state (*k*, *s* − 1) and picked a UMI which was not a new adjacent UMI (i.e., either a previously observed adjacent UMI or not an adjacent UMI). The probability of such a pick is $\left(1-\frac{K-k}{K}{p}_{Adjacent}(U)\right)$.
2. We were previously in a state (*k* − 1, *s* − 1) and picked an UMI which is a new adjacent UMI. The probability of such a pick is $\frac{K-k-1}{K}{p}_{Adjacent}(U)$.

The model above can be evaluated using dynamic programming. To do so we build a matrix *T* = {*t*_*k*, *s*_}, each cell of which contains the weighted sum of the neighboring bottom-left and left cells in the matrix T (see example in Table 3). Such matrices would need to be computed for each UMI present in the dataset. However, the asymptotic complexity of this approach is *O*(*S*_*g*_ ∗  # *UMI* ∗ *K*) in terms of both time and memory, which would be prohibitive for large datasets. To optimize it we employed the following solution. The matrix *T* depends on *U* only through *p*_*Adjacent*_(*U*), and the rate of change of the function within a cell is proportional to *p*_*Adjacent*_(*U*) + *o*(*p*_*Adjacent*_(*U*)). Thus, we assume *p*(*N*′| *S*_*g*_, *U*) to be a piecewise constant function from *p*_*Adjacent*_(*U*) and perform a quantization by this probability. A quantization step Δ*p* = 0.01 was used.

**Table 3:** Dynamic programming matrix with distributions of the number of adjacent UMIs

$\frac{S_g}{K}$	1	2	3	…	*S*_*g*_
0	1	1 − *p*_*Neighb*_	(1 − *p*_*Neighb*_)^2^	...	${\left(1-{p}_{Neighb}\right)}^{S_g-1}$
1	0	*p*_*Neighb*_	$\left(1-{p}_{Neighb}\right)\ast {p}_{Neighb}++{p}_{Neighb}\ast \left(1-{p}_{Neighb}\frac{K-1}{K}\right)$	…	${t}_{0,S-1}\ast {p}_{Neighb}++{t}_{1,S-1}\ast \left(1-{p}_{Neighb}\frac{K-1}{K}\right)$
2	0	0	${p}_{Neighb}^2\frac{K-1}{K}$	…	${t}_{1,S-1}\ast {p}_{Neighb}\frac{K-1}{K}++{t}_{2,S-1}\ast \left(1-{p}_{Neighb}\frac{K-2}{K}\right)$
…	…	…	…	…	…
k	0	0	0	…	${t}_{k-1,S-1}\ast {p}_{Neighb}\frac{K-k+1}{K}++{t}_{k,S-1}\ast \left(1-{p}_{Neighb}\frac{K-k}{K}\right)$
…	…	…	…	…	…
K	0	0	0	…	${t}_{K-1,S-1}\frac{p_{Neighb}}{K}+{t}_{K,S-1}$

Here, K is the maximum number of adjacent UMIs, *S*_*g*_ is the maximum number of molecules per gene. A cell *t*_*k*, *s*_ of the matrix contains probability of observing k adjacent UMIs for a fixed UMI in a cell of size *s*

#### Estimation of the number of erroneous UMIs

To estimate $p\left(\# Error{s}_T=i|\overrightarrow{r_{\Omega_E}},R,{N}_S\right)$ we can assume that an erroneous UMI can occur with some constant probability *p*_*E*_ in each read. Thus, $p\left(\overrightarrow{r_{\Omega_E}}|R, Err\left({\Omega}_E\right)\right)=p\left({r}_E|R, Err\left({\Omega}_E\right)\right)$, where *r*_*E*_ is the total number of reads across all erroneous UMIs: ${r}_E={\sum}_{u^{\prime}\in {\Omega}_E}{r}^{\prime }$. Probability *p*(*r*_*E*_| *R*, *Err*(Ω_*E*_)) was approximated by a binomial distribution with number of trials *n* = *R* + *r*_*E*_. Parameter *p*_*E*_ was estimated using the same training set as for *p*(*q*| *Err*_*U*, *u*_): ${p}_E=\frac{\sum_{g:{S}_g=2}r}{\sum_{g:{S}_g=2}\left(r+R\right)}$. Afterwards, we can estimate distribution of the number of errors as:

$$ p\left(\# Error{s}_T=i|\overrightarrow{r_{\Omega_E}},R,{N}_S\right)=\frac{p\left({r}_i|R, Err\left({\Omega}_E\right)\right)}{\sum_{r\in \overrightarrow{r_{\Omega_E}}}p\left(r|R, Err\left({\Omega}_E\right)\right)}, $$

where *r*_*i*_ is *i*th component of vector $\overrightarrow{r_{\Omega_E}}$.

The problem of estimation of total number of collisions can be formulated as follows: find the distribution of number of distinct UMIs (#*Errors*) after picking #*Errors*_*T*_ UMIs from the pool of all adjacent UMIs. It’s the same problem that we solved when estimating *p*(*N*′| *S*_*g*_, *U*). But in this case probability *p*_*Adjacent*_(*U*) is equal to 1:

$$ p\left(\# Collisions=i|\# Error{s}_T=k\right)=p\left({N}^{\prime }=k-i|{S}_g=k,{p}_{Adjacent}(U)=1\right). $$

### Iterative procedure of UMI sequence error correction

After the estimation of the decision boundary, all UMIs that are determined to be erroneous are removed. This changes the input parameters *S*_*g*_, *N*_*L*_, and *N*_*S*_ of the algorithm. Therefore, to perform a precise filtration, the procedure is run iteratively.This does not add a significant amount of runtime complexity because: i) dynamic programming matrices are calculatd only once, since the gene size cannot increase during filtration; ii) for genes with a small number of UMIs, the procedure converges after one or two iterations.

### Validation

#### UMI trimming

The UMI error correction algorithms become less effective as the number of molecules per gene increases. To model such situations, we used the 10x post-transplant BMMC dataset, which has 10-bp UMIs and relatively small sequencing depth. We then simulated more saturated measurements by trimming UMIs to shorter lengths. The information about each UMI consists of its sequence, the number of reads per UMI, and the mean base-call quality for each nucleotide in the sequence. By trimming both the nucleotide sequence and the quality vector we obtain a new, shorter UMI. After trimming, sequences of some UMIs become identical, which naturally models UMI collision events. All such UMIs are merged by summing their number of reads and calculating the weighted mean of base-call quality vectors (the weight of each vector is equal to its number of reads). For most of the analyses, we trimmed UMIs from the end (back trimming). However, to test for variation of nucleotide diversity along the UMI length, we also trimmed UMIs from the front (see “Results”).

### Distribution of Hamming distances between UMIs of the same gene

Errors in UMI sequences lead to more frequent occurrence of adjacent UMIs. Yet, simply omitting all adjacent UMIs would also be incorrect, as the probability of adjacent UMI occurrence is non-negligible for shorter UMIs and highly expressed genes. Thus, to assess the quality of UMI error correction methods we followed Smith et al. [6] and analyzed distribution of Hamming distances between UMIs within the same gene. To do so we first estimated all pairwise distances between UMIs within each gene within each cell, pooling all distances together. Next, we estimated frequencies of each distance value *P*(*ED* = *k*), and compared it with the theoretical distribution *P*^∗^(*ED* = *k*) of such distances. The theoretical distribution was estimated by random sampling of UMI pairs from a common UMI distribution. The relative difference between the observed distribution and the theoretical one $\left(\frac{\left|P\left( ED=k\right)-{P}^{\ast}\left( ED=k\right)\right|}{P^{\ast}\left( ED=k\right)}\right)$ was compared for different correction algorithms.

### Correction of cellular barcode sequence errors

CB sequence errors split a fraction of the molecules originating from one cell into smaller CBs. Given that the number of reads per UMI is generally higher than one, the smaller CBs will contain some of the same gene–UMI combinations as the true CB. In other words, the smaller CBs will have similar molecular composition—the set of cell unique genes–UMI combinations. We use composition similarity as a criterion for determining whether the two barcodes should be merged. The size of the compositional intersection between two independent cells is modeled using Poisson distribution with the mean dependent on the UMI distribution and the number of molecules associated with the CBs.

Let us denote *S*_*c*, *g*_ as the set of all UMIs detected for gene *g* in a cell *c*. The number of common gene–UMI pairs for cells *i* and *j* can be estimated as ${C}_{i,j}=\sum \limits_{k=1}^m\mid {S}_{i,k}\cap {S}_{j,k}\mid$. Thus, expectation would be $E{C}_{i,j}=E\sum \limits_{k=1}^m\mid {S}_{i,k}\cap {S}_{j,k}\mid =\sum \limits_{k=1}^mE\mid {S}_{i,k}\cap {S}_{j,k}\mid$. The expectation of the UMI intersection (i.e., the number of shared UMIs) for a pair of genes can be estimated as $E{C}_{i,j}=\sum \limits_{u\in UMIs}\left(1-{\left(1-p(u)\right)}^{\left|{S}_{i,k}^{\prime}\right|}\right)\ast \left(1-{\left(1-p(u)\right)}^{\left|{S}_{j,k}^{\prime}\right|}\right)$, where ${S}_{j,k}^{\prime }$ is the number of UMIs in a gene adjusted for UMI collisions. It is important to note that the expected number of shared UMIs needs to be calculated only once for each pair (*S*_*i*_, *S*_*j*_) : *S*_*i*_ ≤ *S*_*j*_. Having estimated *EC*_*i*, *j*_ we can then assume that *C*_*i*, *j*_ follows Poisson distribution with the mean equal to *EC*_*i*, *j*_. Using this estimated distribution, we then perform a statistical test for hypothesis *H*_0_: the observed size of the intersection *S*^∗^_*intersection*_ was obtained by chance. The *P* value of this test is the tail probability of the Poisson distribution ${P}_{\hat{\lambda}}\left({S}_{intersection}\ge {S^{\ast}}_{intersection}|\overrightarrow{E_i},\overrightarrow{E_j},{P}_{UMI}\right)$, where $\hat{\lambda}$ is the estimated intensity parameter.

The implemented pipeline uses this test to compare each cell *C*_*i*_ with all other cells *C*_*j*_ that 1) have a higher total number of molecules (*S*_*j*_ ≥ *S*_*i*_) and 2) whose CBs have a Hamming distance from the CB of *C*_*i*_ that is lower than a fixed constant. In the presented results this distance constant was taken to be 2. Bonferroni correction was used to account for multiple comparisons.

To compare merge algorithms, we evaluated their quality on 10x [10] and Drop-seq [2] human–mouse mixture datasets (datasets 4 and 12) using the following procedure. First, we filtered out all cells that had < 30 genes for 10x and < 20 genes for Drop-seq. Next, for each cell we determined the most likely organism, assigning cells to the genome for which they had more molecules. Next, we chose the largest cells and considered them as real. The exact choice of number of real cells did not have a notable impact on the results. We used 6000 cells for 10x and 1000 cells for Drop-seq. In comparing merge algorithms we counted the number of merges performed between different organisms. Only merges to real cells were counted.

### Classifying damaged or low-quality cells

#### Classification algorithm

The implemented approach for classification of damaged and low quality cells can be split into three tasks: (i) creation of the training sample, i.e., establishing the initial class labeling; (ii) feature selection; and (iii) application of the classifier algorithm.

The initial class labels were assigned based on the cell size. To do so, the dataset was split into three parts: ‘big’ cells, ‘intermediate’ cells, and ‘small’ cells. To determine the borders of big and small cells we used the plot log(#*UMI in cell*) vs log(*cell rank*) (Additional file 1: Figure S10C). This heuristic is based on an observation that the left part of such a plot has a negative second derivative, followed by a linear part, and a third part of the plot has a positive second derivative. We implemented an automated procedure that locates the upper (*t*_*U*_) and the lower (*t*_*L*_) position of the linear part. The cells with size smaller than *t*_*L*_ were then assigned the initial label of ‘low-quality’ cells. The top 75% of the cells with size larger than *t*_*U*_ were assigned the initial label of ‘high-quality’ cells. The remaining cells were labeled as ‘unknown’. Alternatively, the initial label borders can be specified manually, for instance based on the shape of the log(#*UMI in cell* ∗  # *Cells*) vs log(#*UMI in cell*) plots (Additional file 1: Figure S10A, B).

Two types of features could be potentially considered for distinguishing quality cells: biological features (e.g., expression levels of genes belonging to different GO categories [19, 20]), and technical features (e.g., different statistics on the sequenced data). We expect most biological features to be dataset- and cell type-specific [9], with the exception of the mitochondrial fraction, which has appeared as a robust indicator of cell death across most datasets [9]. Therefore, in choosing classifier features we limited consideration of biological features only to the “fraction of UMIs on mitochondrial reads”. The following technical features were also utilized:

1. Mean number of reads per UMI.
2. Mean number of UMIs per gene.
3. Fraction of low-expressed genes (genes with one molecule).
4. Fraction of intergenic reads.
5. Fraction of not-aligned reads (optional feature, as it typically has to be calculated during the identifier parse phase).

Given the initial labeling and the feature set, the cell classification problem was considered as a problem of establishing robust classification in the presence of training label noise [21]. We compared three classifiers: Kernel Density Estimation classifier [12], Random Forest [13], and Robust Gaussian Processes Classifier [14]. Following evaluation of robustness we chose the KDE classifier with Normal Scale bandwidth selector [22] (the implementation provided by the R package ‘ks’ was utilized [23]). The computational complexity of the KDE classifier estimation has exponential dependency on the dimensionality of the feature space. We therefore reduced the feature space by using the first three principal components of the feature space for classification. To increase the algorithm robustness, we used sparse robust principal component analysis [24] (R package ‘pcaPP’) with sparsity level *λ* = 1.

The algorithm’s performance can be improved by labeling cells with very high fractions of mitochondrial and intergenic reads as ‘low-quality’. This can be done prior to classifier training, or simply as an additional filter after classifier training. To choose between these two options we employed the following condition: if the intergenic or mitochondrial fraction contributes to any of the first three PCs with the loading ≥5%, we assume that the algorithm is able to distinguish between high/low fraction values, and labeling for the corresponding fraction can be done prior to the classifier training. Otherwise, labeling is done after the training. The extreme fraction thresholds we determined as *m* + 4*a*, where *m* is the 20% trimmed mean mitochondrial (or intergenic) fraction across cells in the dataset, and *a* is the median absolute deviation of the corresponding fraction.

### Validation of the results

Validation of the algorithm was based on the assumption that the rescued cells (i.e., cells with low numbers of molecules, which would be filtered with size-threshold-based algorithms) should have similar gene expression patterns to the real cells. As a first step, KDE classification was performed for all cells that passed a pre-defined threshold on the minimal number of expressed genes. The threshold value was taken to be 20 for the inDrop datasets and 50 for the larger 10× 8k PBMC dataset. Cells that had a quality score less than 0.9 and the number of molecules less than *t*_*U*_ were filtered out (omitted). Next, we annotated cell types and performed differential expression analysis for each type. We selected several cell types (i.e., cell clusters) that showed substantial cell differences between the threshold-based and KDE filtration, and generated gene expression heatmaps for all cells in these clusters, showing the most differentially expressed genes for each cluster (see “Results”). To plot such gene expression heatmaps we: (i) normalized molecule counts for each gene by the total number of molecules detected in a given cell; (ii) transformed expression values to their rank values within a gene; and (iii) normalized by the total number of cells on the plot (to obtain values in the [0; 1] range). A similar procedure was used for t-SNE visualization of gene expression in Additional file 1: Figures S12–S14. To choose cluster-specific genes we used the following procedure:

1. For each cluster we identified differentially expressed genes by comparing it against every other cluster using the Seurat R package [25].
2. For each differential gene we counted the number of clusters where it was detected. No more than 50 genes with the largest number of clusters were picked.
3. Only genes that were expressed in > 60% of the cells in at least one of the clusters were shown.

### Mouse bone marrow inDrop measurements

Whole bone marrow cells were isolated from 11-week-old C57Bl/6 male mice (Jackson Laboratory). The epiphysis/metaphysis fraction from long bones was collected, crushed, cut into small pieces, and digested using Collagenase I (STEMCELL Technologies) with agitation for 30 min at 37 °C. Bone marrow cells were filtered through a 70-μm filter. Red blood cells were lysed using Ack-lysis (ThermoFisher Scientific) on ice for 5 min, quenched with Media 199 (ThermoFisher Scientific) supplemented with 2% fetal bovine serum (ThermoFisher Scientific), and spun down at 500 g for 5 min. Cells were stained for 30 min with the red blood cell marker TER119 (Biolegend) and cells were sorted using DAPI (ThermoFisher Scientific) as a live/dead viability marker. Live whole bone marrow cells (400,000; negative for TER119) were sorted into medium 199 (ThermoFisher Scientific). Before inDrop encapsulation cells were counted using a Cellometer (Nexcelom Bioscience). Cell viability was over 90%.

#### InDrop processing

The concentration of cells was adjusted to 300,000 cells/ml by adding PBS to the sorted cells. The cell suspension was then mixed 1:1 (v/v) with PBS containing 30% OptiPrep Density Gradient Medium (Sigma D1556) to obtain 150,000 cells/ml in 15% Optiprep. Using four microfluidics pumps and a polydimethylsiloxane (PDMS) microfluidic device, about 10,000 cells were co-encapsulated with barcoded polyacrylamide beads and a reverse transcription mixture containing Superscript III into water-in-oil droplets, according to a published protocol [26]. The library preparation and quality control procedures were carried out as described [26]. Indexed libraries were pooled and sequenced on a Next-seq 500 system (Illumina) at 2 pM concentrations.

### Mouse–human cell line mixture inDrop measurement

CK1750 mouse lung cancer cells (Carla Kim laboratory, Boston Children’s Hospital) and K562 human immortalized myelogenous leukemia cells (ATCC) were mixed at a 1:1 ratio to obtain 70,000 cells/ml in PBS containing 15% Optiprep. About 3000 cells were co-encapsulated with barcoded polyacrylamide beads and a reverse transcription mixture containing Superscript III into water-in-oil droplets; and a library was prepared according to a published protocol [26]. The library was sequenced on a MiSeq system (Illumina).

## Availability and requirements

**Name:** dropEst

**Homepage:** https://github.com/hms-dbmi/dropEst

**OS:** linux, OS X

**Programming language:** C++, R

**License:** GPL-3.

## Additional files

Additional file 1: Supplementary figures with legends. (PDF 4377 kb)

Additional file 2: Supplementary tables. (PDF 41 kb)

Additional file 3: Example of the report, generated by the pipeline. (PDF 544 kb)
