# Supplementary material for: dropEst: pipeline for accurate estimation of molecular counts in droplet-based single-cell RNA-seq experiments
Source: Genome Biol. 2018 Jun 19;19:78. doi: 10.1186/s13059-018-1449-6 (PMC6010209; doi:10.1186/s13059-018-1449-6)
Supplement: Supplementary file 1 — Supplementary figures with legends. (PDF 4377 kb) [file 13059_2018_1449_MOESM1_ESM.pdf]

Supplementary Figure Legends

dropEst: pipeline for accurate estimation of molecular counts in droplet-based single-cell RNA-seq experiments

Viktor Petukhov[1,2], Jimin Guo[2], Ninib Baryawno[3,4,5], Nicolas Severe[3,4,5], David T. Scadden[3,4,5], Maria G. Samsonova[1], Peter V. Kharchenko[2,3,4]

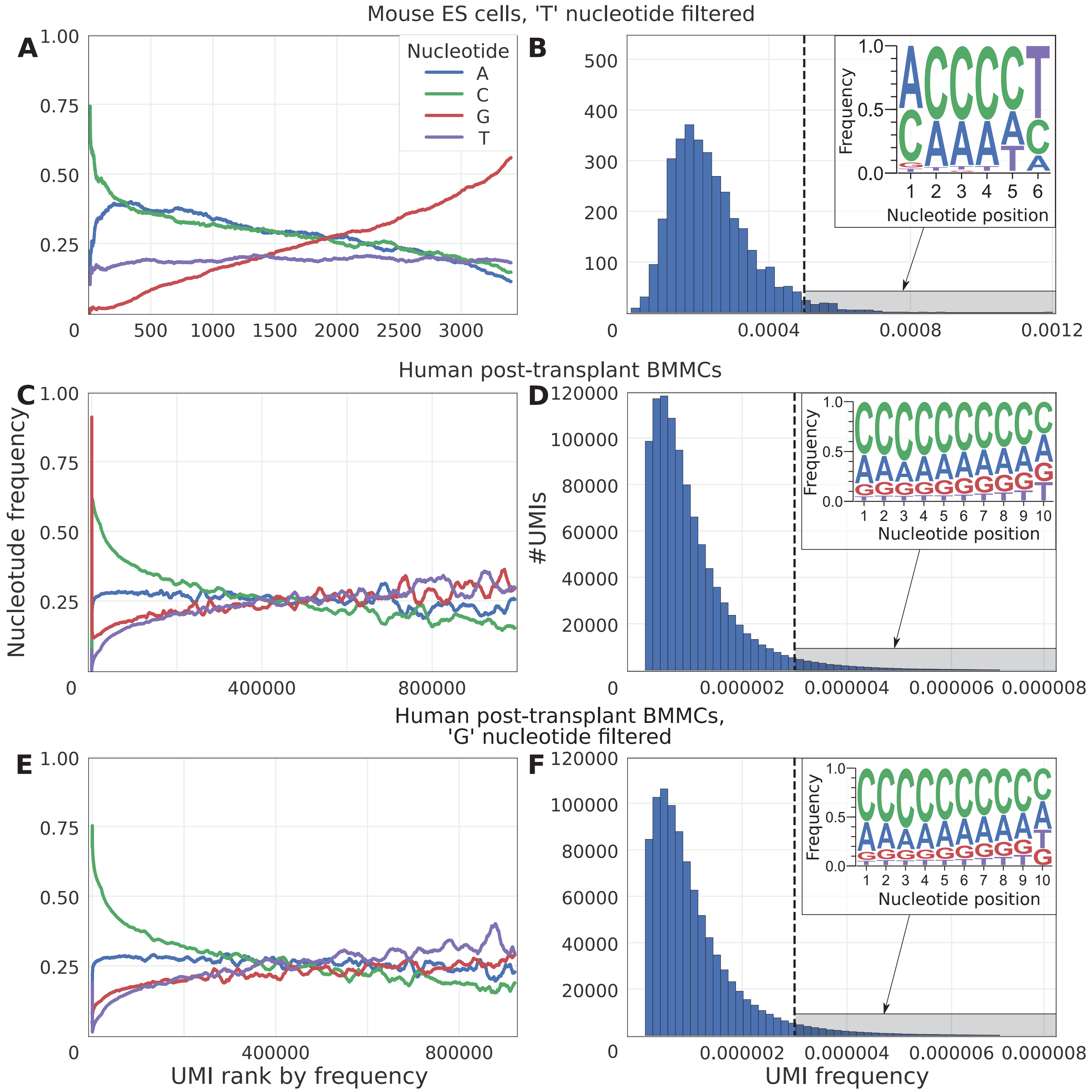

Figure S1. Skewness of UMI distributions.

As in Figure 1 of the main manuscript, nucleotide frequencies and UMI distributions are shown for the inDrop mouse ES cells dataset (dataset 1) after the filtration of extreme right tail (A, B). The analogous plots are also shown for 10x the human post-transplant BMMCs dataset before (C, D) and after the filtration (E-F). The filtering only has a visible effect on the left peak of the distribution, and does not affect the overall skewed shape of the distribution.

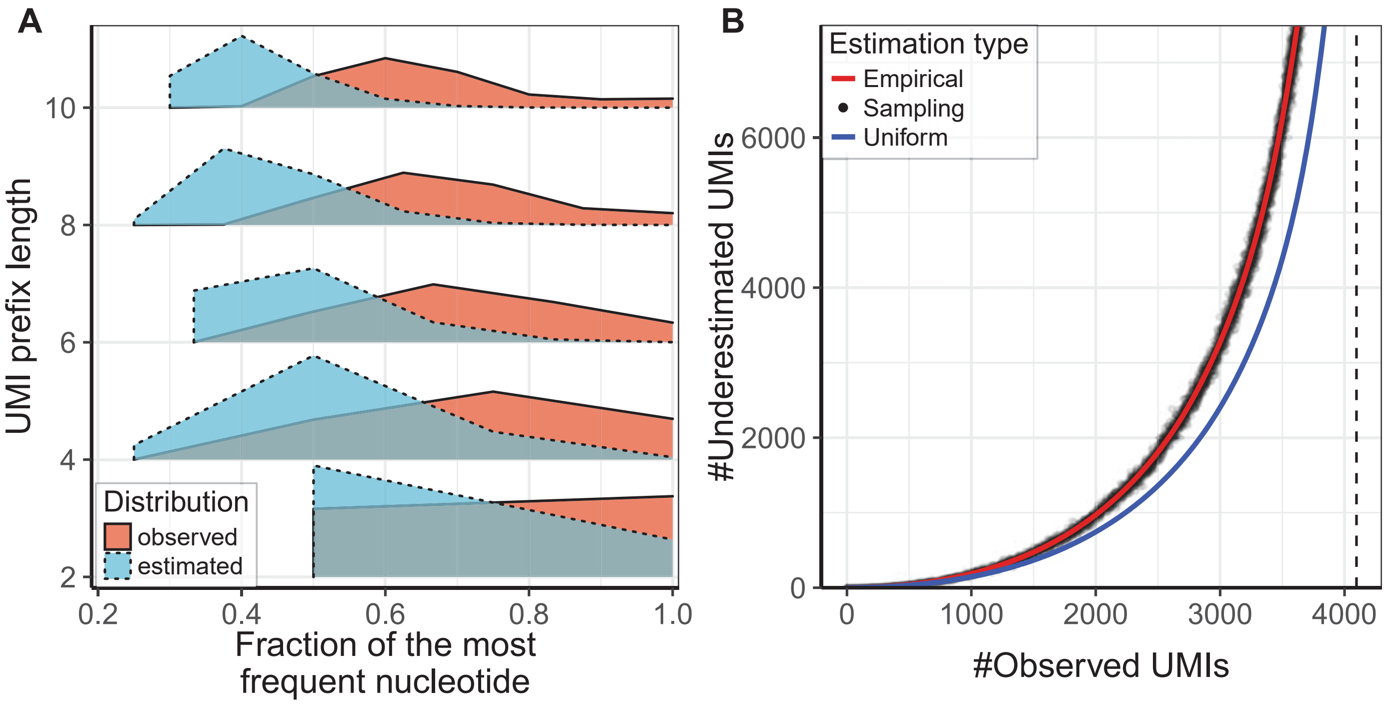

Figure S2. Simulation of UMI collision frequencies.

(A) Many of the UMIs forming the extreme right tail of the UMI frequency distribution have low nucleotide diversity. We took the most frequent 0.5% of the UMIs (4582 sequences) and calculated distribution of the fraction of the most frequent nucleotide (x axis) over these UMIs. This distribution is shown for the UMI prefix subsequences of different lengths, from the first 2bp to the whole 10bp UMI (different ridges on the y axis). "Estimated" distribution was obtained by randomly sampling UMIs from the full UMI distribution, and averaging the most common nucleotide frequency across multiple sampling rounds. 10x post-transplant BMMC dataset (dataset 7) was used.

(B) To evaluate the developed approach for correcting UMI collisions, we modeled collisions by randomly sampling UMIs from observed distribution using mouse BMC dataset (dataset 11, see Methods). The number of the resulting unique UMIs after the merge is shown on the x axis. The y axis shows the number of collisions (*i.e.* the difference between the total number of unique molecules before and after the merge). The observed number of collisions in artificial (sampled) genes is shown with black dots. The collision frequencies predicted by different models are shown by lines.

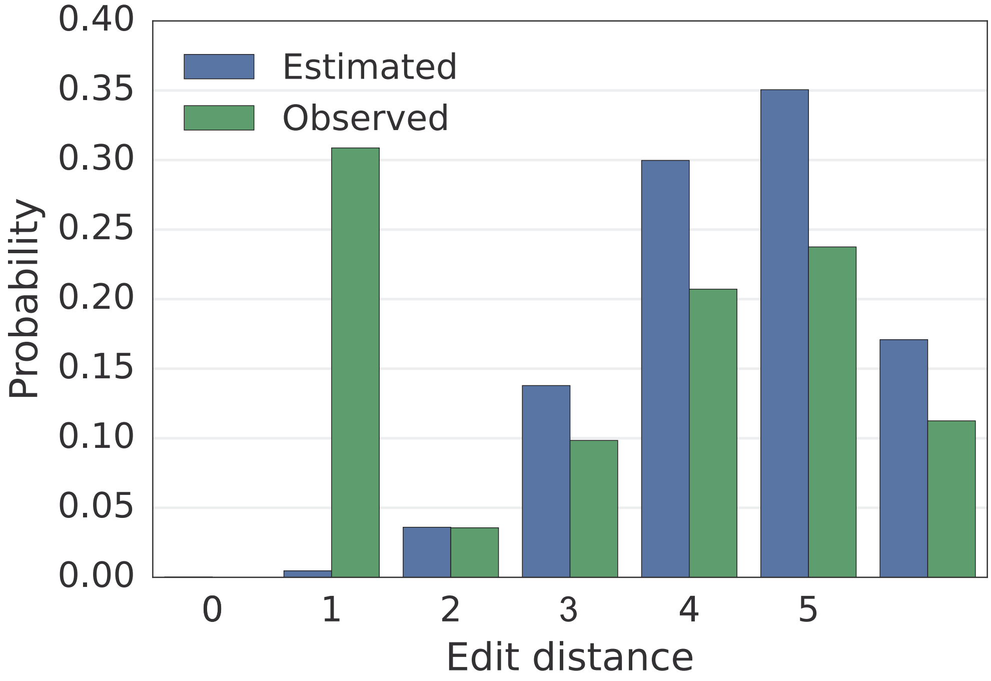

Figure S3. Probability of observing adjacent UMIs in small genes.

Probability of observing two adjacent UMIs in a given gene in a given cell (y axis) was aggregated by all cells and genes is shown for different edit distances (x axis). The probability estimates based on the bootstrap procedure are shown in blue. The empirically observed probabilities are shown in green. Mouse ES dataset (dataset 1) was used. The observed number of adjacent UMIs exceeds the expected estimates by a factor of 40, indicating that most of the adjacent UMI occurrences result from UMI sequence errors.

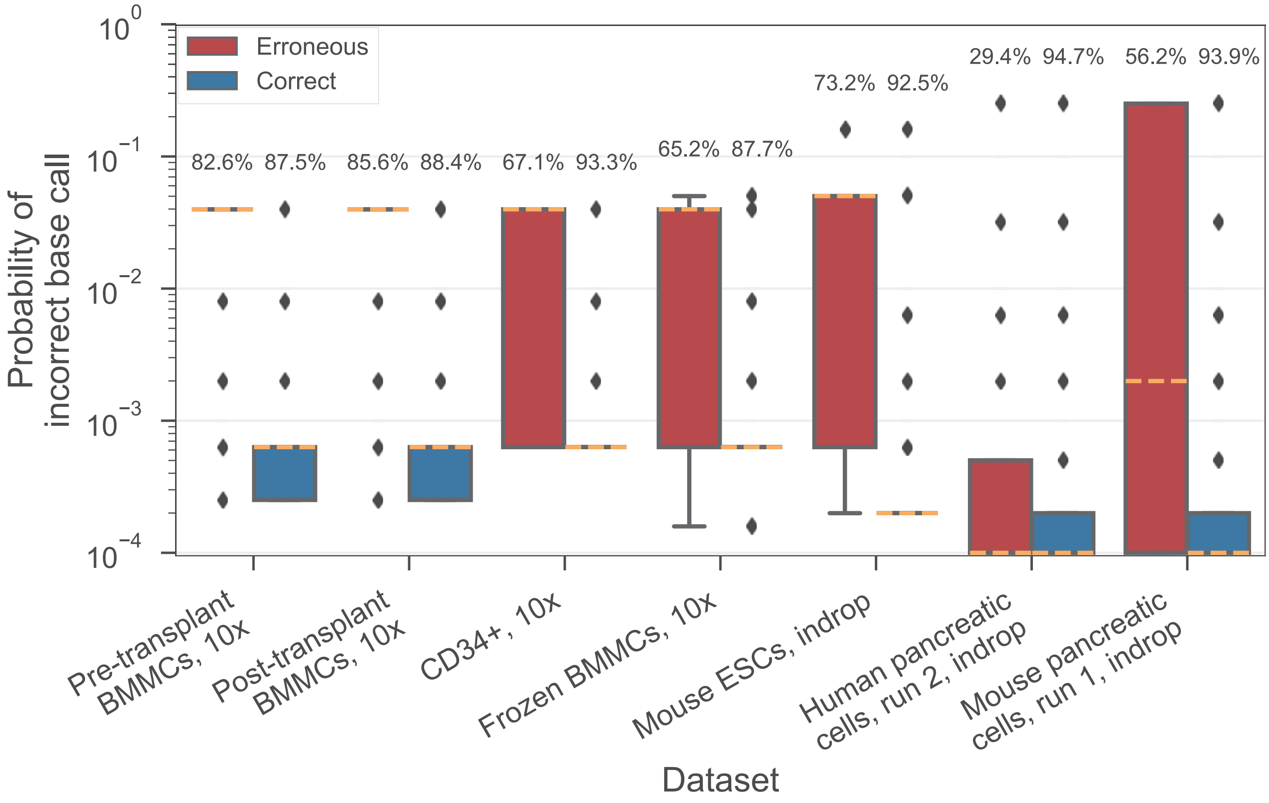

Figure S4. Recognition of UMI errors by base calling quality.

Errors in UMI sequence can occur during either amplification or sequencing. Depending on the source, these errors may or may not be distinguished by the base call quality. To examine the contribution of these mechanisms to the UMI sequence errors we compared distributions of base call quality in different subsets of data. We focused on the adjacent UMIs (same gene, same cell), most of which are expected to be erroneous (Figure S3). The boxplots show for each dataset, the distribution of the nucleotide base call quality for the adjacent (red) and distant (blue) UMIs. Specifically, the red box plots show base call quality for the nucleotide discrepant between the adjacent UMIs. The blue box plots show quality distribution across all nucleotides in pairs of UMIs that are sufficiently distant (Hamming distance > 3). The numbers above the box plots show the fraction of reads of each type that were correctly classified based on the base call quality alone (using naive Bayes classifier). The results suggest that the fraction of UMI errors that can be easily explained by the base call quality varies between the datasets, ranging from 29.4% to 85.6%.

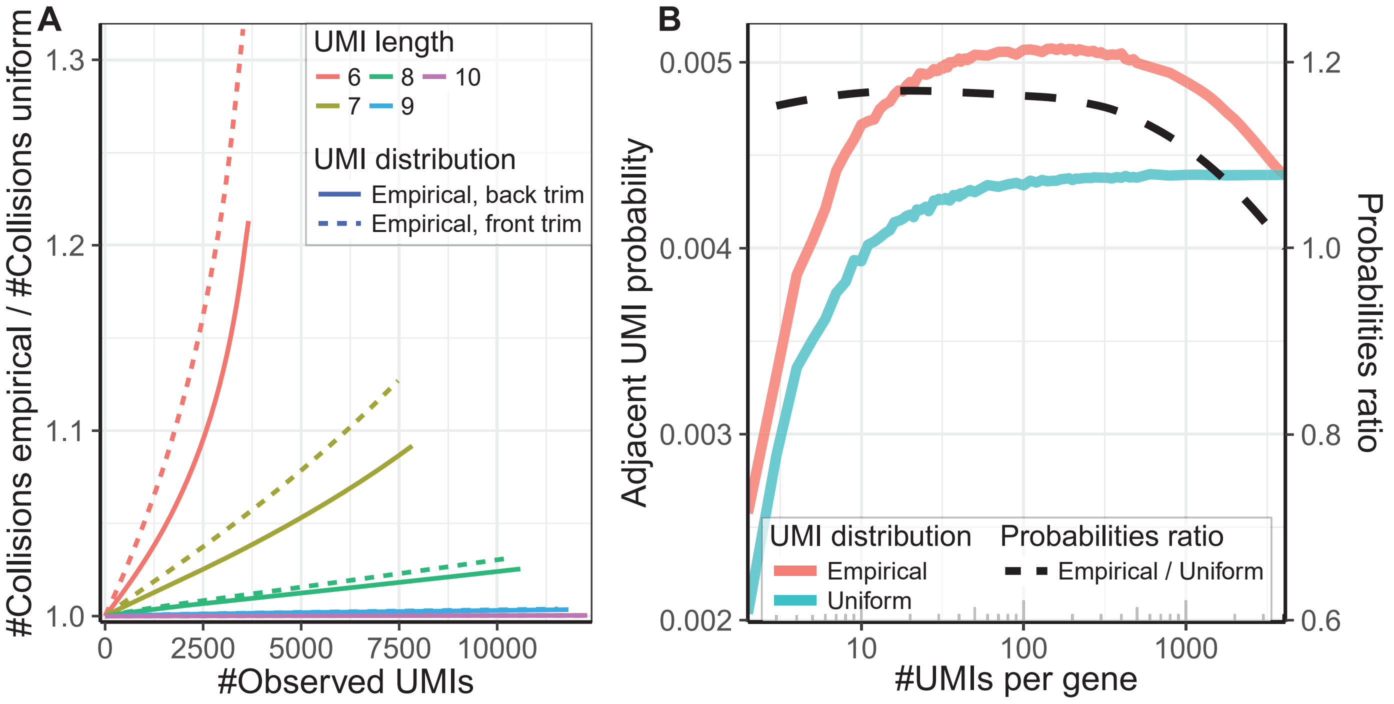

Figure S5. Impact of non-uniform distribution on UMI collisions.

(A) An alternative presentation of the Figure 1C of the main manuscript is shown. The ratio between the two methods of collision adjustment (y axis) is shown as a function of the true gene expression level (x axis) for two types of UMI trimming simulations: trimming from the front and from the back of the UMI sequence (see Methods). Front trimming leads to the larger difference between the two methods, due to larger skewness of the resulting trimmed UMI distribution (i.e. lower sequence complexity).

(B) The probability of observing a pair of adjacent UMIs (left y axis) depends on the number of molecules per gene (x axis). Such probabilities are shown for the two estimates of the UMI distribution: uniform and empirical. The ratio of these two distributions is shown by the dashed line (right y axis). Here, inDrop mouse BMCs dataset (dataset 11) was used.

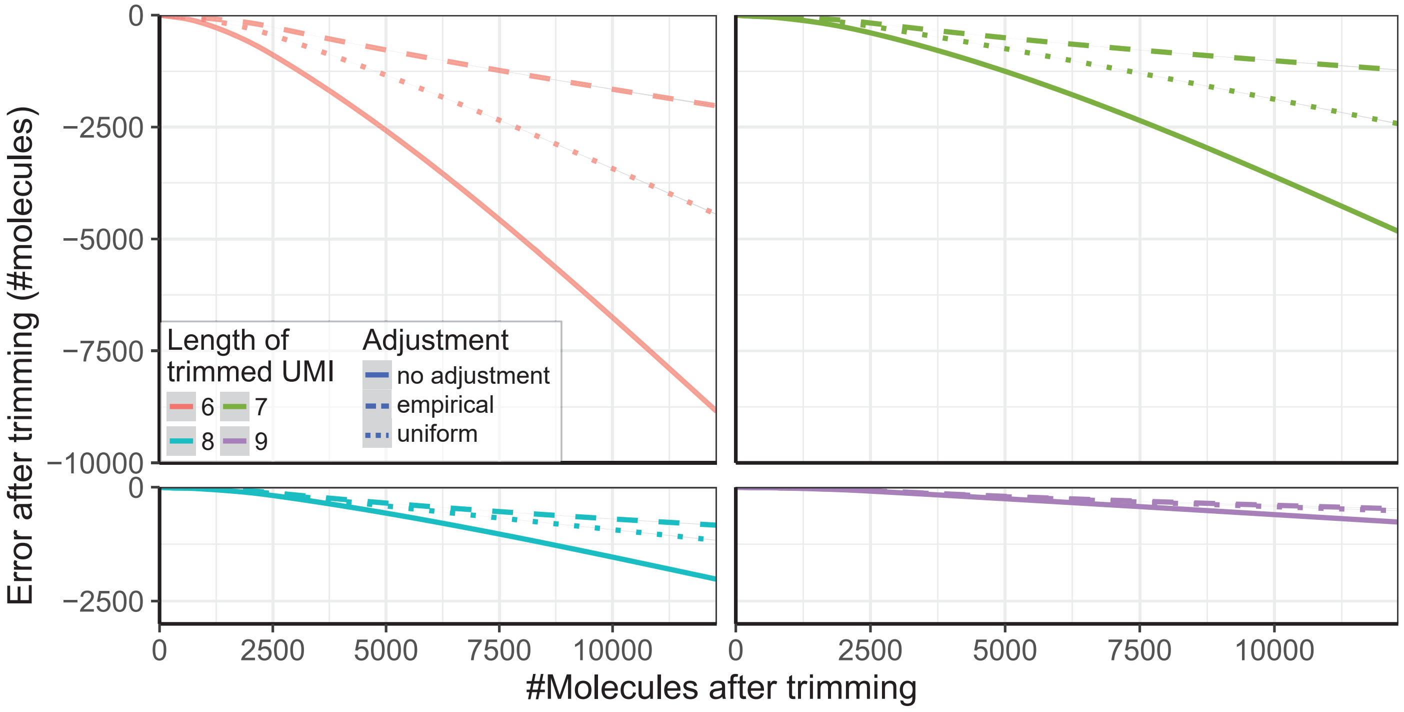

Figure S6. UMI collisions on trimmed data.

The plots show effect of different UMI collision adjustment approaches on the trimmed UMI data (dataset 7). The true number of molecules (estimated on untrimmed UMIs with *cluster* correction, see Methods) is shown on the x axis, with the y axis showing the difference between the observed and the true number of molecules for different lengths of trimmed UMIs. The lines were obtained using spline smoothing, as in Figure 2 of the main manuscript.

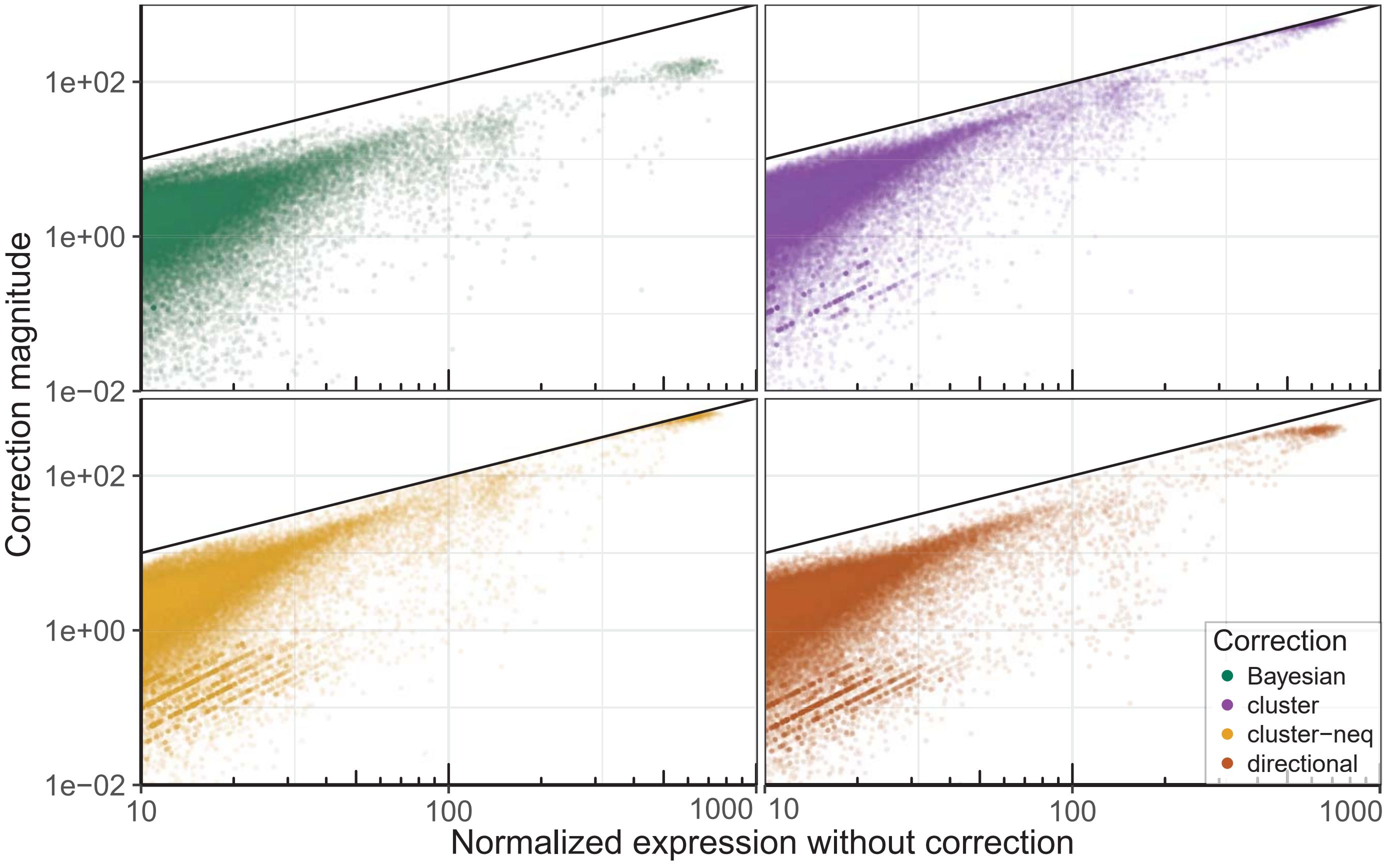

Figure S7. Magnitude of UMI correction.

Similar to Figure 2E of the main manuscripts, the plots show dependency of the magnitude of UMI correction (y axis) on the expression magnitude, calculated prior to correction (x axis).

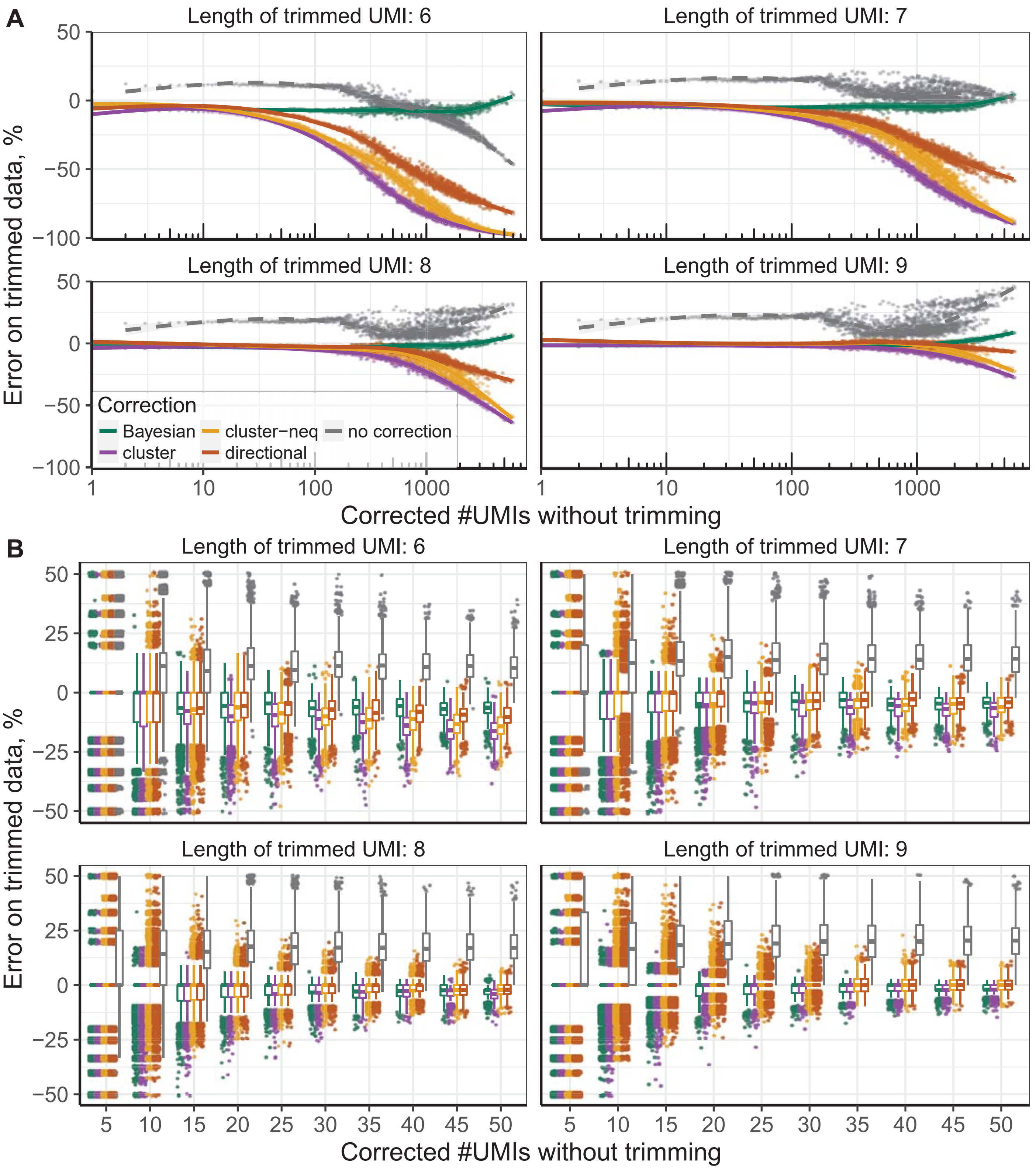

Figure S8. Comparison of UMI correction algorithms on trimmed data.

(A) Similar to Figure 2C of the main manuscript, comparison of UMI correction algorithms is shown on the 10x post-transplant BMMC dataset (dataset 7). The x axis shows the estimated true magnitude, based on full-length UMIs with *cluster* correction (see Methods). The y axis shows the observed error of different methods on trimmed UMI data relative to the true magnitude. The UMI collisions were corrected using *empirical* approach in all cases except for "no correction". The length of trimmed UMIs is given by labels above each plot.

(B) The boxplots show the same data as **(A)**, restricted to moderately expressed genes only. Gene expression magnitudes were aggregated across all cells and grouped by the nearest multiple of 5. The position of the outlier points is shown using horizontal jitter.

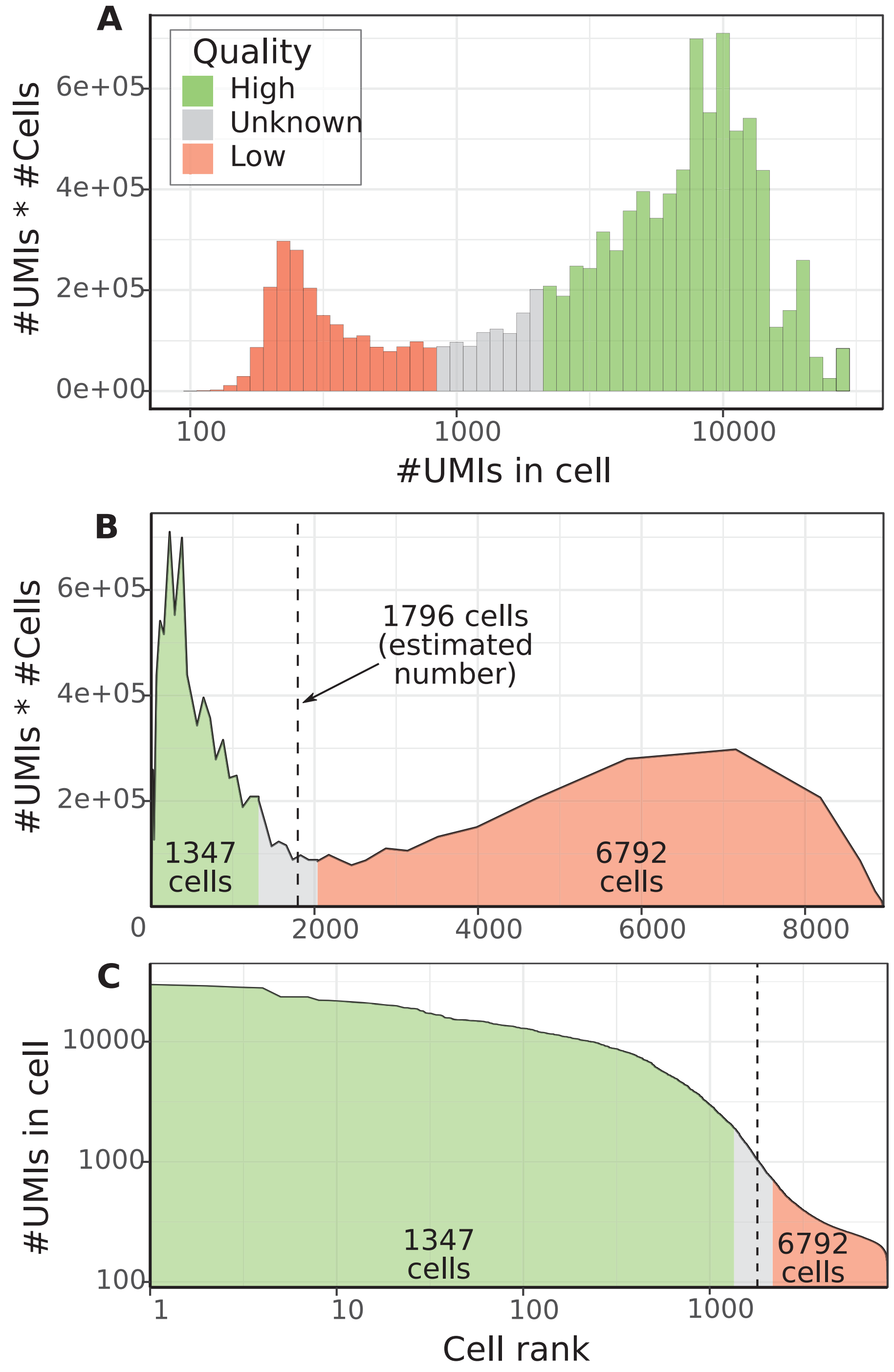

Figure S9. Initial labeling of high-quality cells based on cell size distributions.

Three heuristic ways of assigning initial cell labels to high-quality and low-quality cells are shown.

(A) Shows scaled histogram, where the cells were binned based on the total number of molecules. For each bin, the y axis gives the number of molecules multiplied by the number of cells, so that the overall plot shows the distribution of molecular counts as a function of cell size. Such distributions tend to be bi-modal and the local minimum can be used to estimate the separation between the well-measured cell population (right) and low-quality/empty droplets (left). However, such pattern is not apparent in some datasets.

(B) Similar pattern can be obtained by changing the x axis to map molecular counts on the cell rank (with the largest cell being assigned the lowest rank).

(C) An alternative approach is to identify the size threshold based on the second derivative of the cumulative size distribution plot (see Methods). Here the y axis gives the total size of the cell, and x axis orders the cells by rank. The cells within the 25% of the identified thresholds (by rank) are initially labeled as Unknown (see Methods).

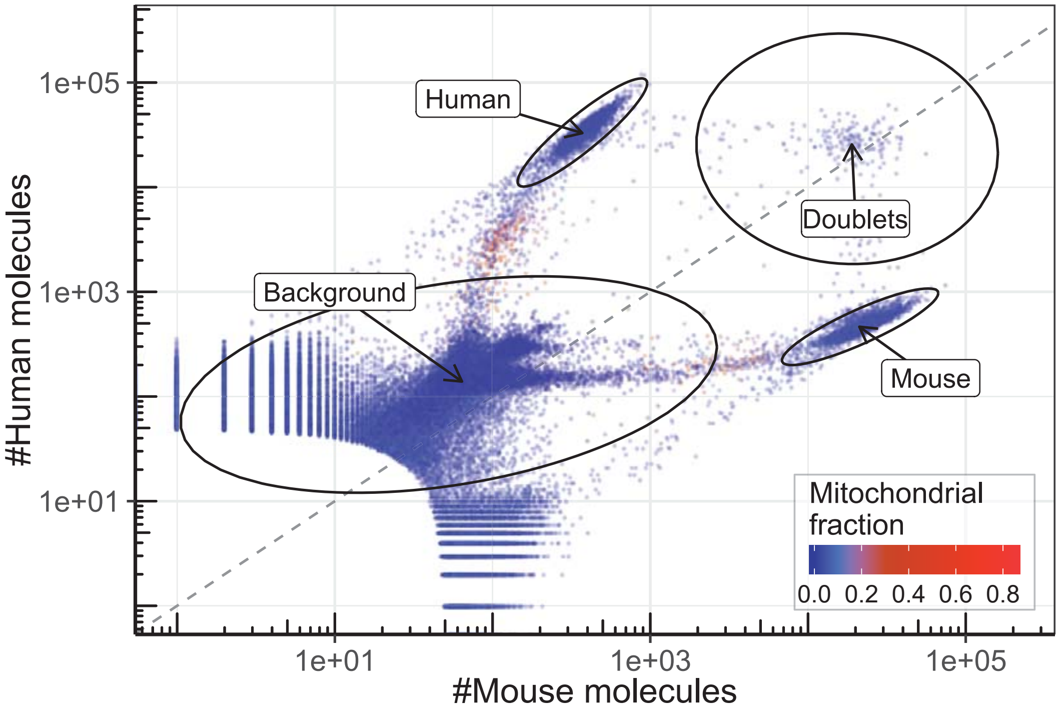

Figure S10. Human and mouse cell mixture dataset by 10x.

Analogous to Figure 3A of the main manuscript, the plot shows number of molecules mapped to human and mouse genomes for the CBs observed in the 10x human / mouse mixture data (dataset 4).

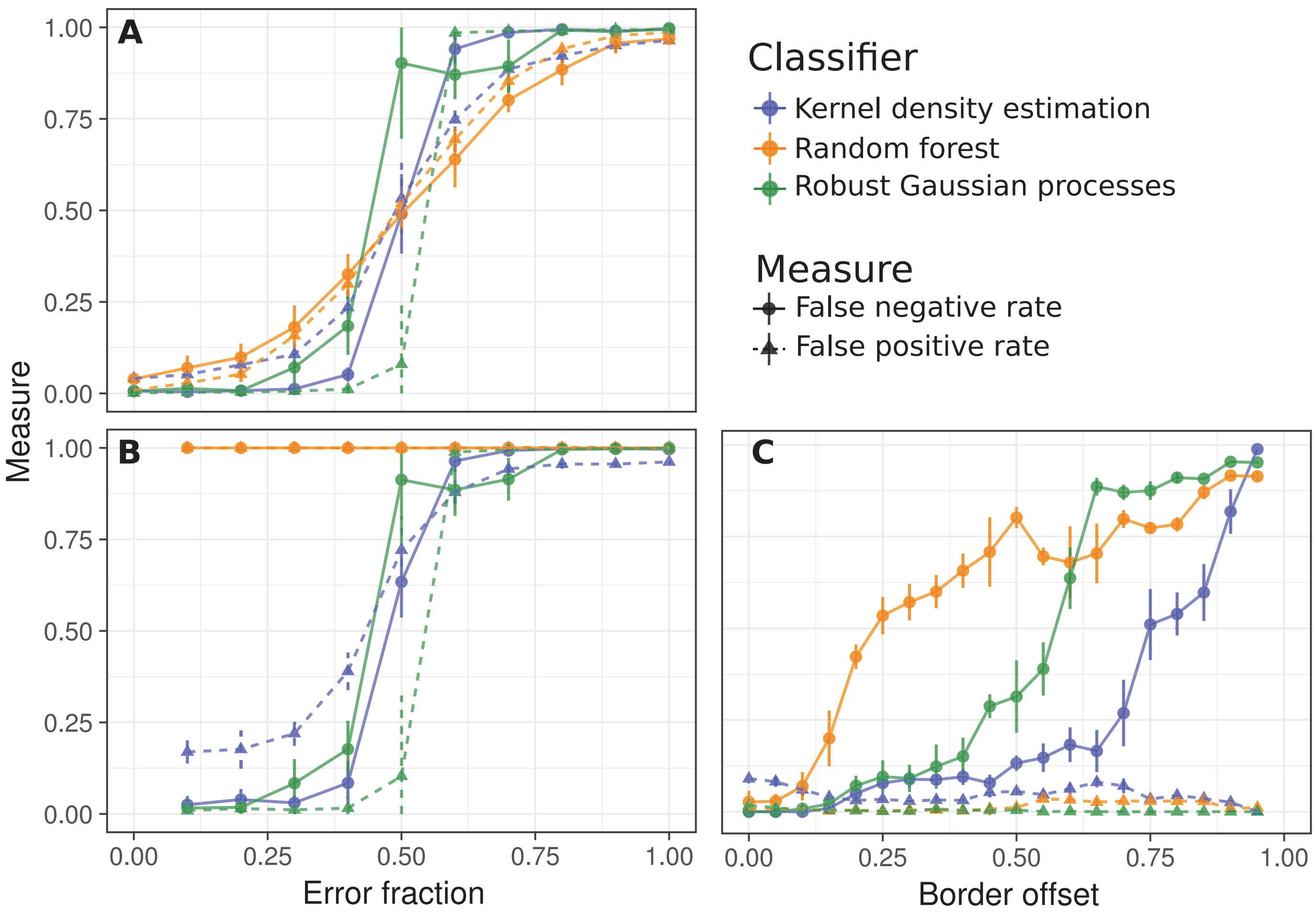

Figure S11. Robustness of different classifiers to training errors.

(A) As classification of low-quality cells depends on the ability to tolerate high level of errors in the initial training label assignment, we examined the robustness of different types of algorithms to artificially-added errors. Specifically, each classifier was first trained on the initial data, and the resulting labels were used for subsequent perturbations (to avoid simulation bias). Then 80% of the data was used to re-train the classifier, but a fixed percentage of class labels (x-axis) were swapped. The plot shows false positive and false negative rates (y axis) of different classifiers on the remaining 20% of the data. Here, the inDrop Mouse Pancreatic Cells dataset, run 1 (dataset 3) was used.

(B) Analogous plot shows performance of the classifiers on the labels that were swapped in the 80% of the data that was used for training.

(C) Robustness of the classifiers to the initial labeling thresholds. The cell size borders used to assign the initial labels were widened by a fixed percentage (x axis), and the performance of the classifiers was compared using the labels, which the classifiers were able to predict based on the original borders.

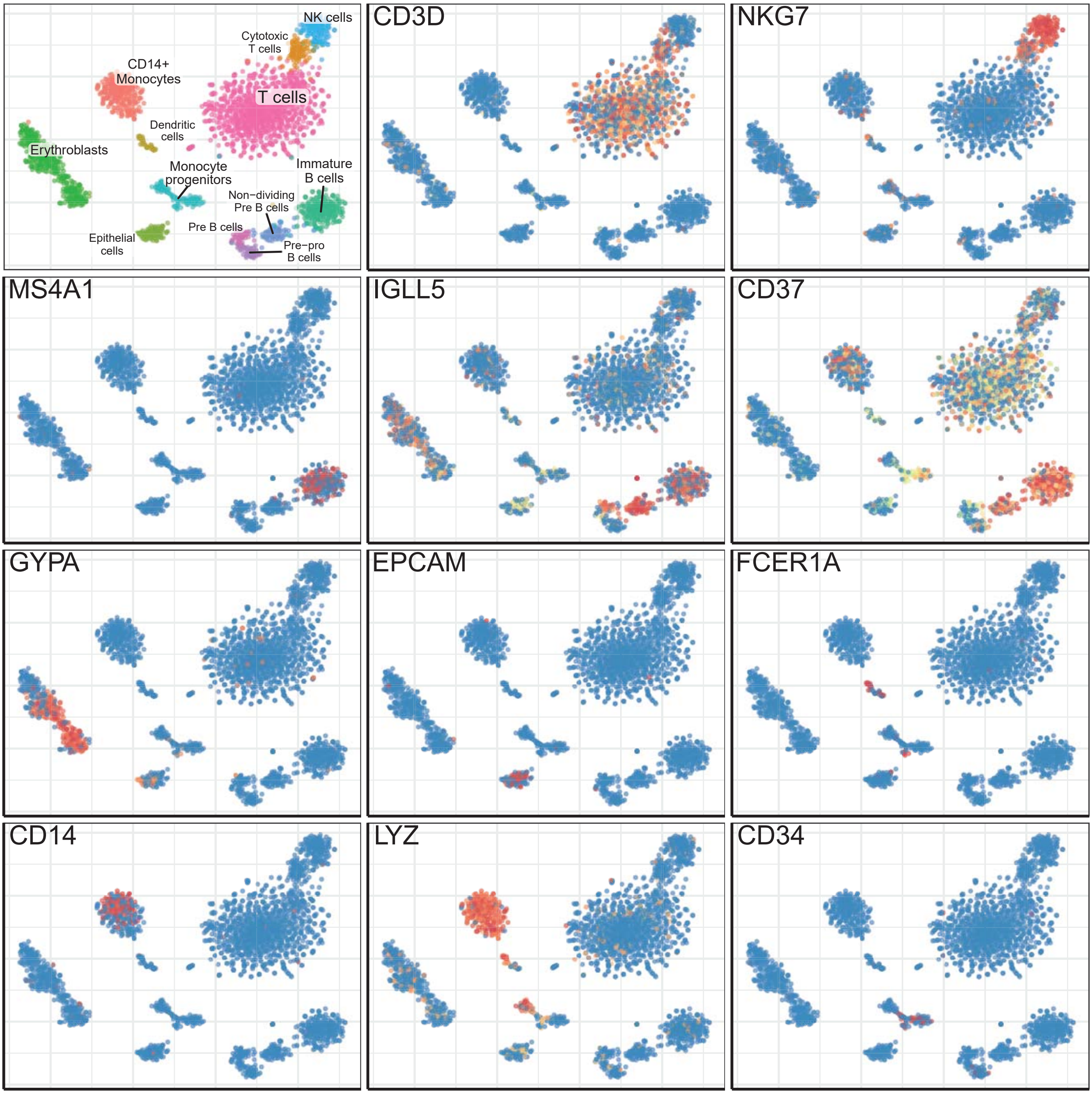

Figure S12. Annotation of the 10x Frozen BMMCs dataset.

The figure shows expression of genes, which were used for cell type annotation, on t-SNE visualization of the 10x Frozen BMMCs dataset (dataset 8). The first subplot shows complete annotation, and the following subplots show expression values of the corresponding genes. Gene names are indicated in the top-left corner of each plot. For better visualization, gene expression values were rank-normalized, similarly to the heatmaps on the Figures 4-6 of the main manuscript (see Methods for details).

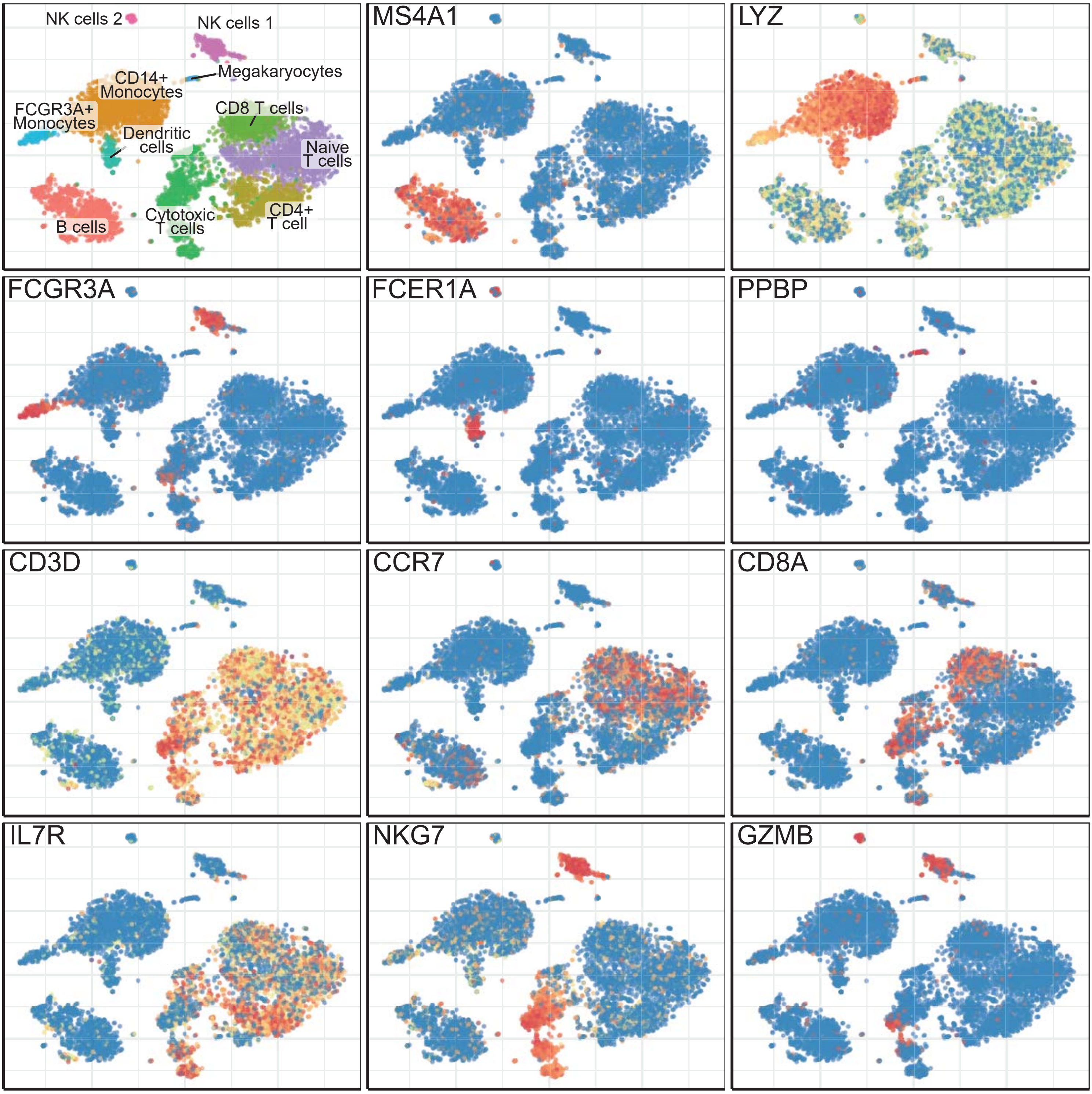

Figure S13. Annotation of the 10x 8k PBMCs dataset.

Similar to Figure S12, annotated cell populations are shown for the 10x 8k PBMCs dataset (dataset 13).

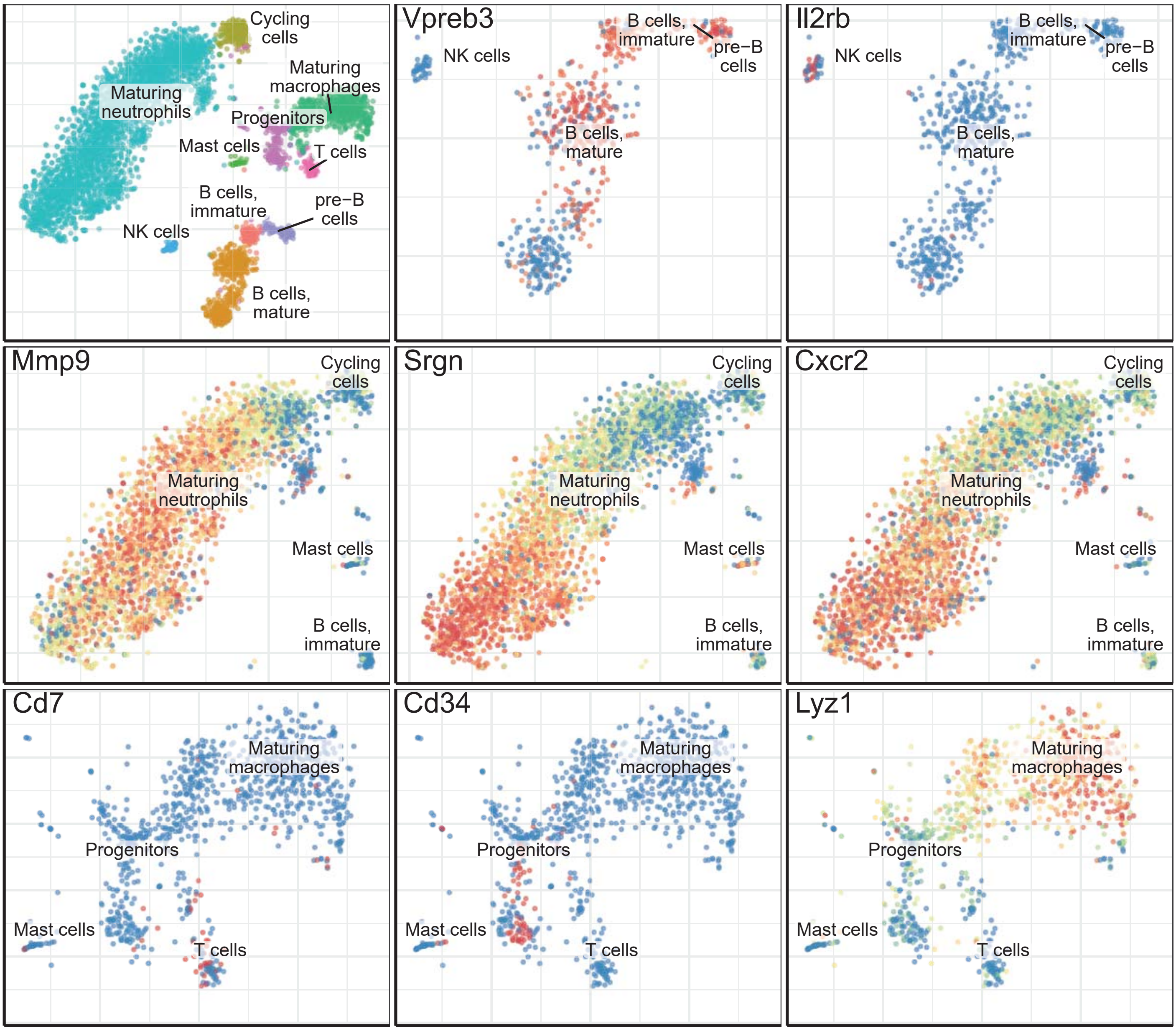

Figure S14. Annotation of the inDrop BMCs dataset.

Similar to Figure S12, annotated cell populations are shown for the inDrop BMCs dataset (dataset 11). Different panels zoom into different parts of the overall t-SNE embedding (top-left panel) so that smaller subpopulations can be clearly labeled.

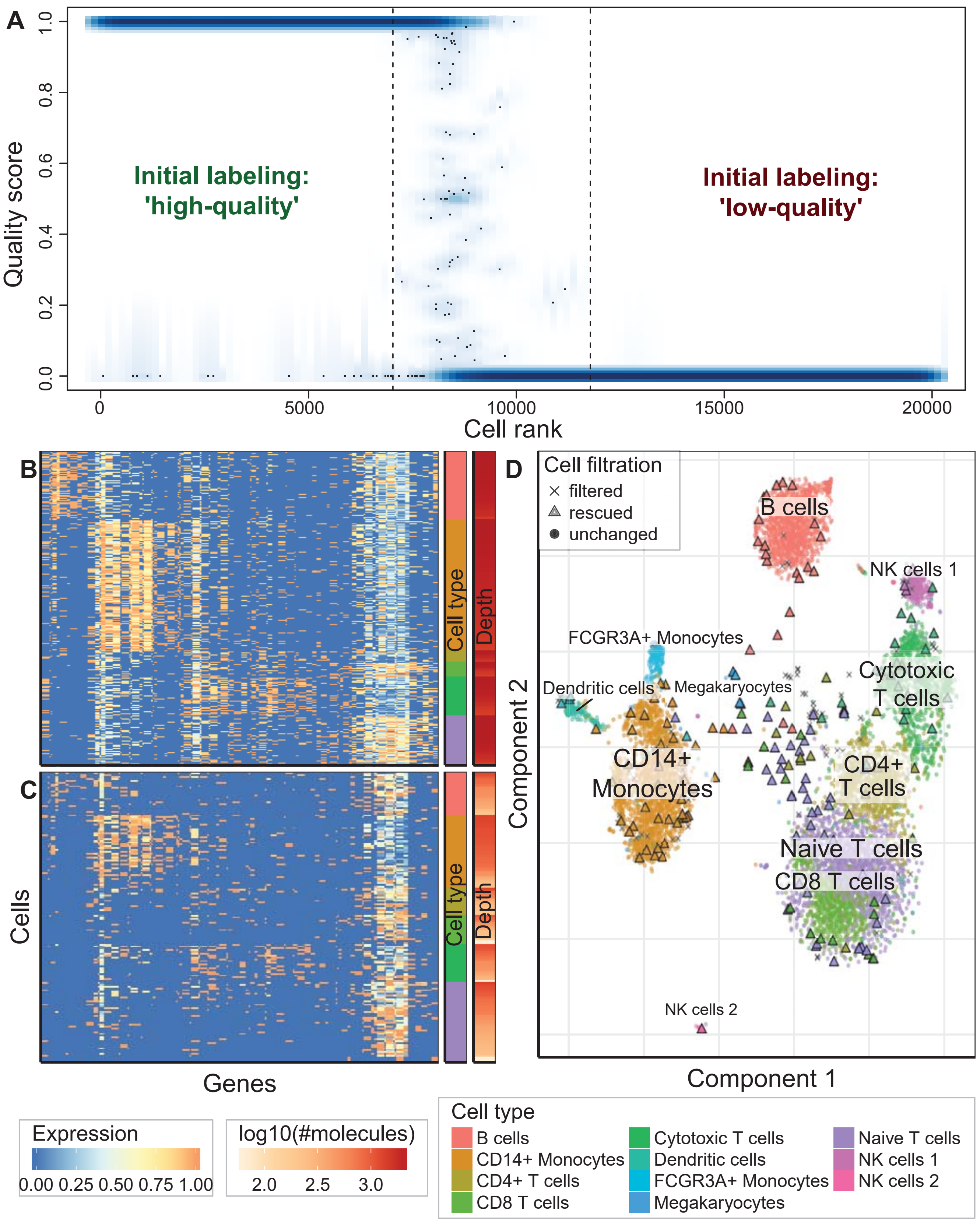
A
Quality score
1.0
0.8
0.6
0.4
0.2
0.0
Initial labeling: 'high-quality'
Initial labeling: 'low-quality'
0
5000
10000
15000
20000
Cell rank
B
C
Cells
Genes
Cell type
Depth
D
Cell filtration
filtered
rescued
unchanged
B cells
NK cells 1
FCGR3A+ Monocytes
Dendritic cells
Megakaryocytes
Cytotoxic T cells
CD14+ Monocytes
CD4+ T cells
Naive T cells
CD8 T cells
NK cells 2
Component 2
Component 1
Expression
0.00 0.25 0.50 0.75 1.00
log10(#molecules)
2.0 2.5 3.0
Cell type
B cells
CD14+ Monocytes
CD4+ T cells
CD8 T cells
Cytotoxic T cells
Dendritic cells
FCGR3A+ Monocytes
Megakaryocytes
Naive T cells
NK cells 1
NK cells 2

Figure S15. Classification of low- and high-quality cells on 10x data.

(A) Cell quality score (y axis), as determined by the developed approach is shown as a function of the cell size (x axis, shown as rank, with the largest cells being assigned the lowest rank) for 10x 8k PBMCs dataset (dataset 13). The initial size-group assignment of each cell is shown by colored labels. As expected, most large cells are reported to have high scores, and cells with small number of molecules tend to have low quality scores. Nevertheless, some of the large cells are classified as being of low in quality and some of the small cells are classified to have high quality.

(B) Heatmap shows expression of cluster-specific genes for the ‘high-quality’ cells, as determined by both KDE-based and size threshold-based algorithms.

(C) Same genes are shown for the cells “rescued” by the KDE-based algorithm (i.e. cells with high quality scores that were below the initial size threshold). The heatmaps are similar to the Figure 6A,B of the main manuscript. See Methods for additional details.

(D) t-SNE visualization of the 10x 8k PBMCs data (dataset 13), similar to Figure 6C of the main manuscript. In this plot, cells were selected according to the 10x Cell Ranger cell size filtration threshold.

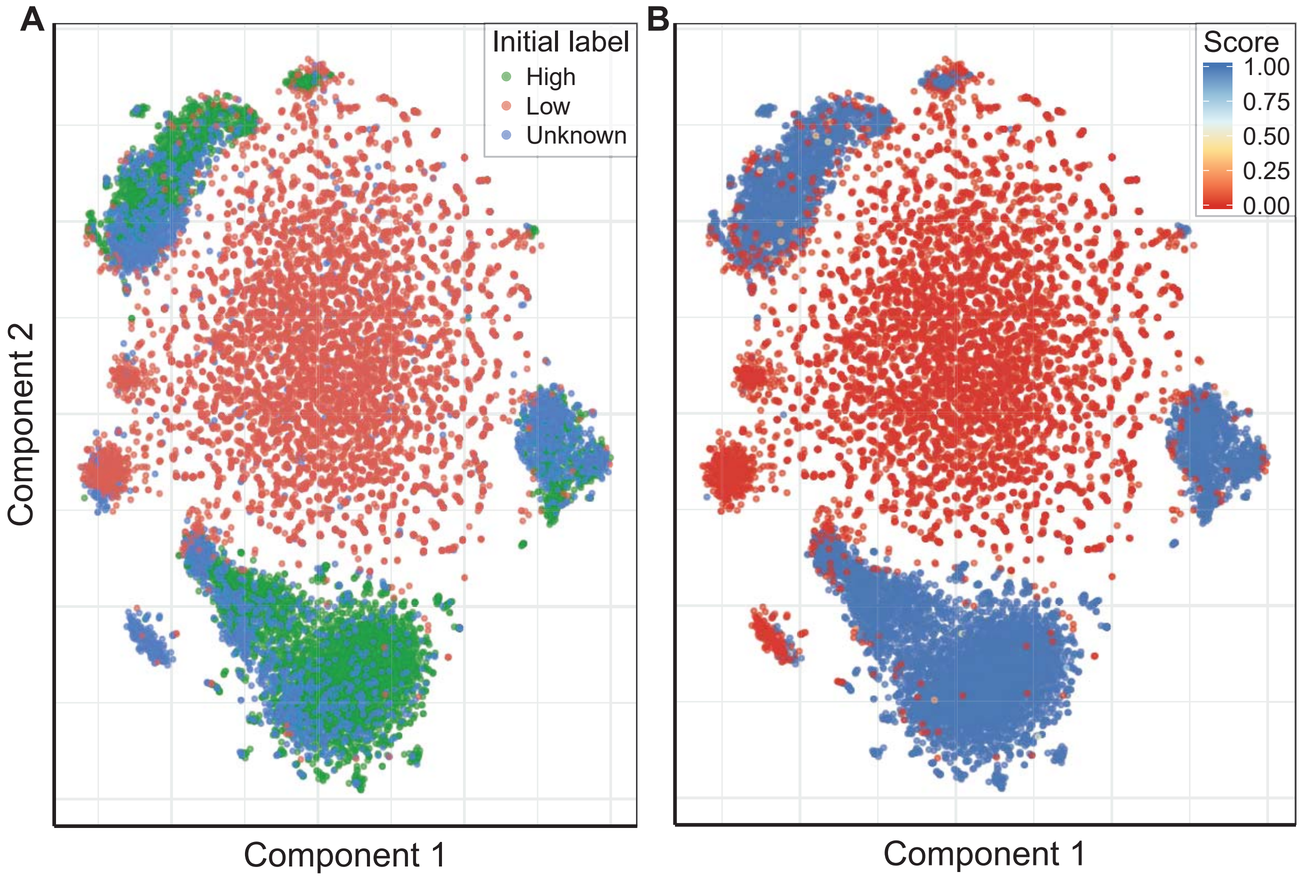

Figure S16. Comparison of the initial label assignments with the cell quality score predicted by the algorithm.

t-SNE visualization shows all 10x 8k PBMCs (dataset 13) colored according to: **(A)** their initial (size-based) quality labels, and by **(B)** quality score determined by the KDE classification algorithm. Overall, the algorithm recognizes lower-size high-quality cells that are found within the major population clusters. Conversely, almost all of the cells outside of the major clusters are classified to have low (<0.5) quality scores.

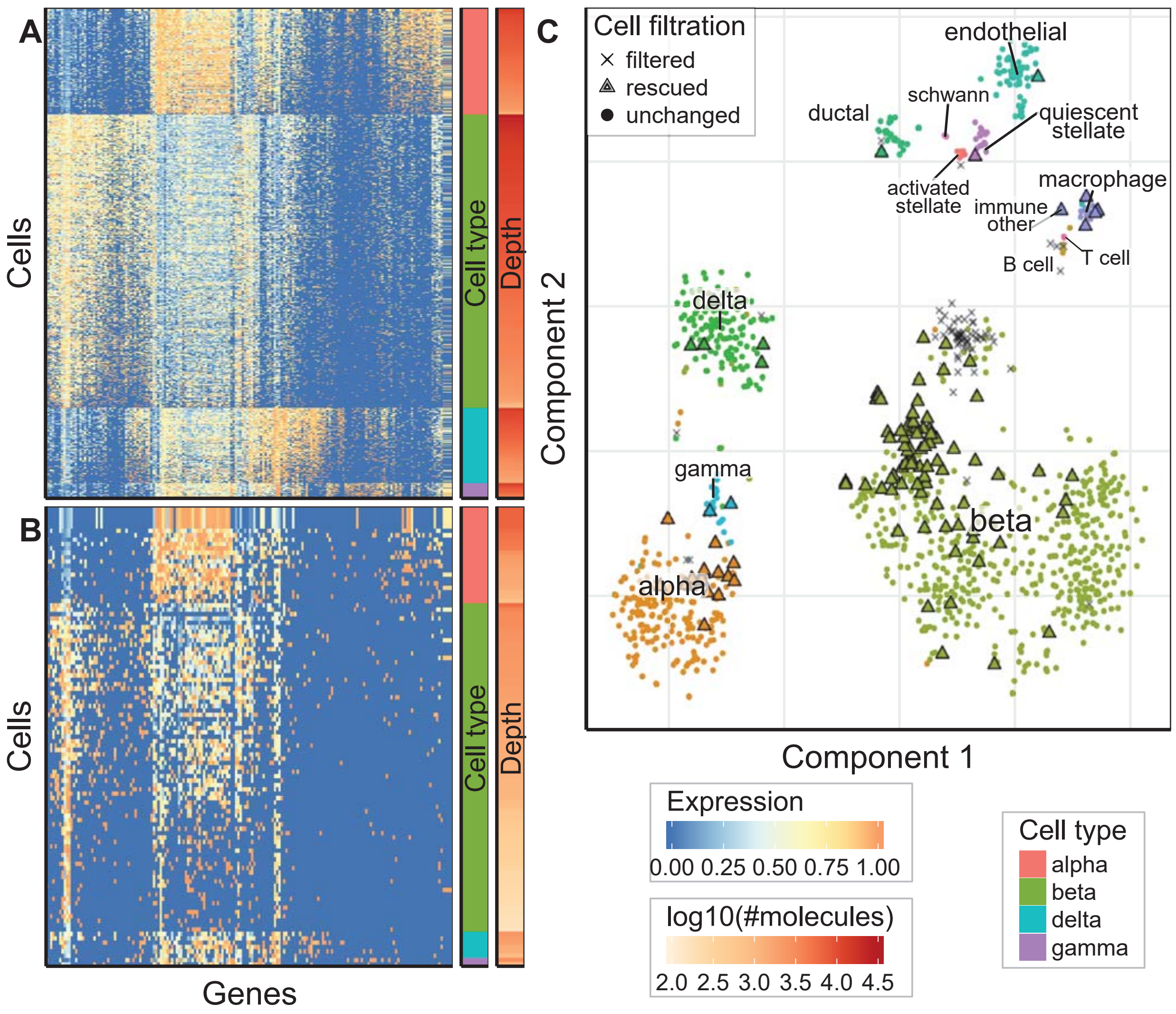

Figure S17. Classification of low- and high-quality cells on inDrop mouse pancreatic cells data.

Figure, similar to Figure 5, for the inDrop mouse pancreatic cells data (dataset 3). Cell annotations from the original paper were used.
