# Supplementary material for: dropEst: pipeline for accurate estimation of molecular counts in droplet-based single-cell RNA-seq experiments
Source: Genome Biol. 2018 Jun 19;19:78. doi: 10.1186/s13059-018-1449-6 (PMC6010209; doi:10.1186/s13059-018-1449-6)
Supplement: Supplementary file 2 — Supplementary tables. (PDF 41 kb) [file 13059_2018_1449_MOESM2_ESM.pdf]

Table S1. Gene markers, used for annotation of the 10x Frozen BMMCs.

Cell Type	Markers
B cells	CD19, MME, MS4A1
T cells	CD3D
CD14+ Monocytes	LYZ, CD14
NK cells	GZMA, GZMB, GNLY, NKG7
Dendritic cells	FCER1A, CST3
Monocyte progenitors	LYZ, CD34
Epithelial cells	EPCAM, CD226-, APOE
Erythroblasts	GYPA, CD36, TFRC
Cytotoxic T cells	NKG7, GZMA, GZMH, GZMK
T cells	CD3E, CD8B, IL7R
Immature B cells	MS4A1, CD40, IL4R, IL7R-
Pre-pro B cells	CD34, CD38, MME, CD24-, IL7R-
Pre B cells	CD34-, CD40-, IL7R+, IL4R-, CD19+
Non-dividing Pre B cells	CD34, IGLL5

Table S2. Gene markers, used for annotation of the 10x 8k PBMCs dataset.

Cell Type	Markers
B cells	MS4A1
T cells	CD3D, CD3E
CD14+ Monocytes	LYZ, CD14
NK cells 1	GNLY, NKG7
NK cells 2	GZMB, NKG7
FCGR3A+ Monocytes	FCGR3A, MS4A7
Dendritic cells	FCER1A, CST3
Megakaryocytes	PPBP
Naive T cells	CCR7
CD8 T cells	CD8A, CD8B
CD4+ T cells	IL7R, CD4
Cytotoxic T cells	NKG7, GZMA, GZMH, GZMK

Table S3. Gene markers, used for annotation of the inDrop mouse BMCs dataset.

Cell Type	Markers
Maturing neutrophils	Mmp9, Srgn, Cxcr2
Maturing macrophages	Cd14, Ly6c2
T cells	Cd7
B cells	Cd79a, Cd79b
B cells, mature	Cd74, Cd83
B cells, immature	Vpreb1
pre-B cells	Vpreb3
Progenitors	Cd34, Kit
NK cells	Nkg7, Il2rb, Thy1

Table S4. Fraction of rescued cells for the 10x Frozen BMMCs dataset.

Cell type	Total num. of cells	Num. of rescued	Fraction of rescued, %
CD14+ Monocytes	305	164	53.77
Cytotoxic T cells	105	37	35.24

Dendritic cells	90	16	17.78
Epithelial cells	135	22	16.3
Erythroblasts	510	264	51.76
Immature B cells	277	133	48.01
Monocyte progenitors	157	33	21.02
NK cells	179	47	26.26
Non-dividing Pre B cells	93	82	88.17
Pre B cells	76	8	10.53
Pre-pro B cells	65	30	46.15
T cells	1098	269	24.5

Table S5. Fraction of rescued cells for the 10x 8k PMMCs dataset.

Cell type	Total num. of cells	Num. of rescued	Fraction of rescued, %
B cells	1239	23	1.86
CD14+ Monocytes	1833	40	2.18
CD4+ T cells	956	15	1.57
CD8 T cells	972	16	1.65
Cytotoxic T cells	1153	21	1.82
Dendritic cells	224	4	1.79
FCGR3A+ Monocytes	197	0	0
Megakaryocytes	44	5	11.36
Naive T cells	1452	42	2.89
NK cells 1	322	4	1.24
NK cells 2	69	2	2.9

Table S6. Fraction of rescued cells for the inDrop mouse PCs dataset.

Cell type	Total num. of cells	Num. of rescued	Fraction of rescued, %
activated stellate	10	0	0
alpha	199	18	9.05
B cell	8	0	0
beta	623	75	12.04
delta	137	6	4.38
ductal	41	2	4.88
endothelial	68	1	1.47
gamma	29	2	6.9
immune other	4	0	0
macrophage	24	5	20.83
quiescent stellate	18	1	5.56
schwann	3	0	0
T cell	3	0	0

Table S7. Fraction of rescued cells for the inDrop mouse BMCs dataset.

Cell type	Total num. of cells	Num. of rescued	Fraction of rescued, %
B cells, immature	99	0	0

B cells, mature	457	1	0.22
Cycling cells	192	5	2.6
Mast cells	48	1	2.08
Maturing macrophages	653	1	0.15
Maturing neutrophils	2876	113	3.93
NK cells	61	0	0
pre-B cells	120	0	0
Progenitors	222	4	1.8
T cells	82	1	1.22

Table S8. Runtimes of dropEst pipeline.

**Dataset**	**Phase**	**Can be parallel**	**Size**	**#Cores**	**Time**
InDrop Mouse BMCs	dropTag	Yes	244759580 reads	1	1h 14m
	dropEst	No	69420268 reads	1	27m
	CB Merge	No	78282 CBs	1	9m 40s
	UMI Collisions	No	7619389 UMIs	1	10.0s
	UMI Errors	Yes		10	621.1s
	UMI Errors “directional”	Yes		10	38.5s
	Quality scoring	No	6536 cells	1	11.5s
InDrop Mouse ES Cells	dropTag	Yes	413138104 reads	1	1h 25m
	dropEst	No	184395185 reads	1	38m
	CB Merge	No	90168 CBs	1	11m 20s
	UMI Collisions	No	13738402 UMIs	1	10.4s
	UMI Errors	Yes		10	879.4s
	UMI Errors “directional”	Yes		10	51.2s
	Quality scoring	No	6234 cells	1	4.7s

Here, processor Intel(R) Xeon(R) CPU E5-2697 v3 @ 2.60GHz was used.
