# Supplementary material for: dropEst: pipeline for accurate estimation of molecular counts in droplet-based single-cell RNA-seq experiments
Source: Genome Biol. 2018 Jun 19;19:78. doi: 10.1186/s13059-018-1449-6 (PMC6010209; doi:10.1186/s13059-018-1449-6)
Supplement: Supplementary file 3 — Example of the report, generated by the pipeline. (PDF 544 kb) [file 13059_2018_1449_MOESM3_ESM.pdf]

Supplementary Note 1: Pipeline report example

The subsequent pages shows pipeline report (originally HTML) for the Human CD34+ 10x dataset.

Common info

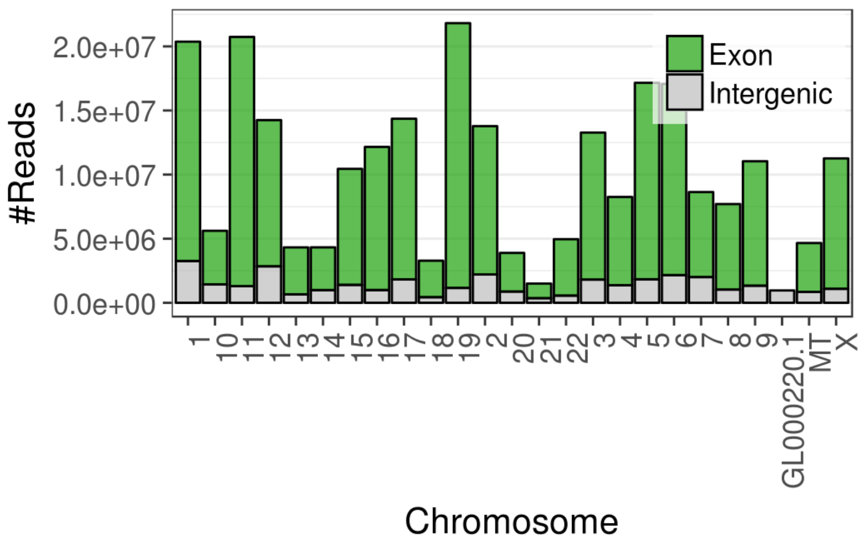

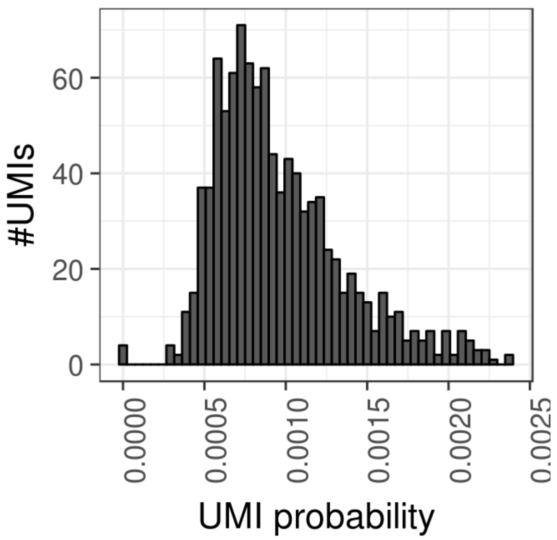

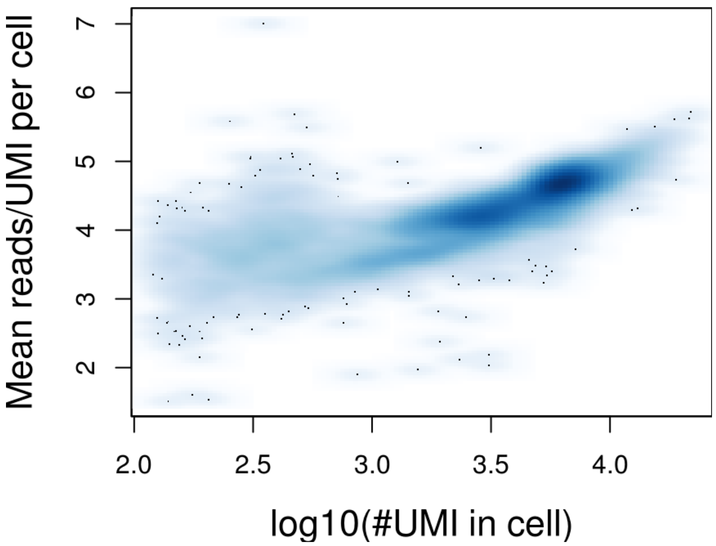

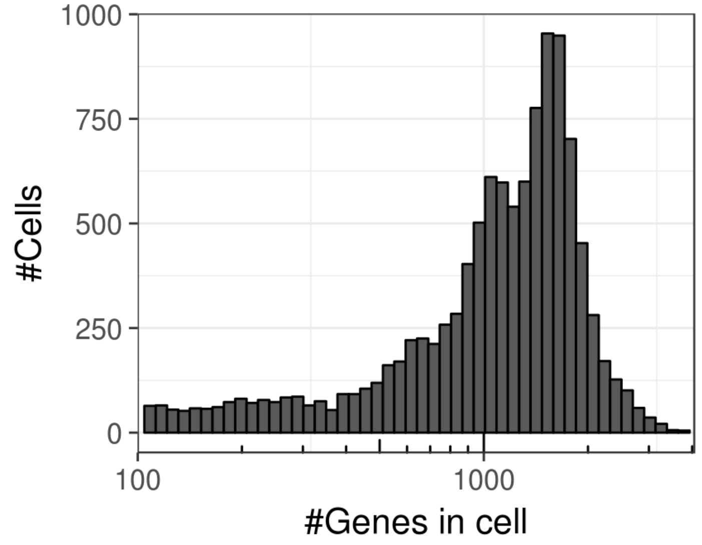

Number of cells

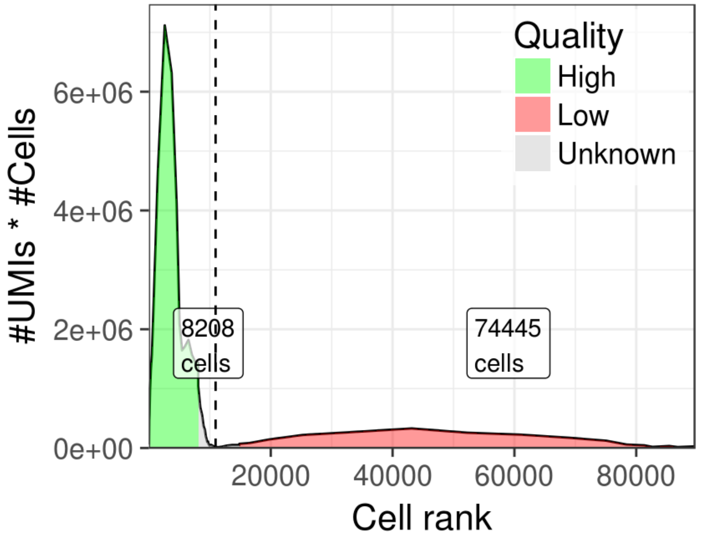

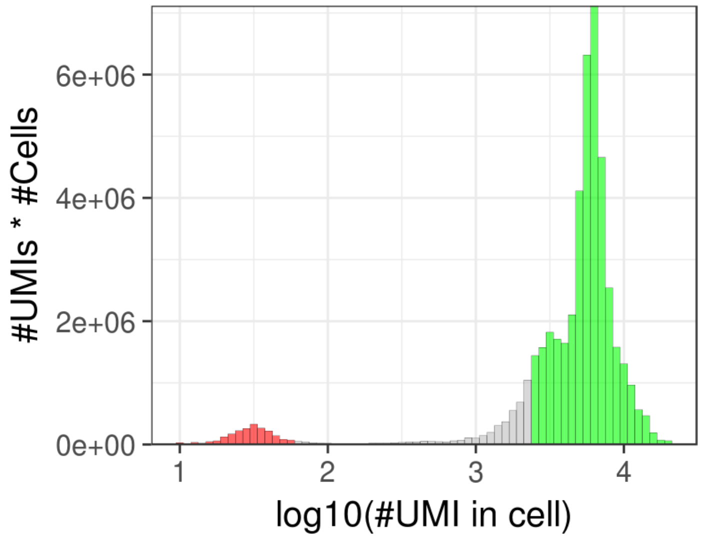

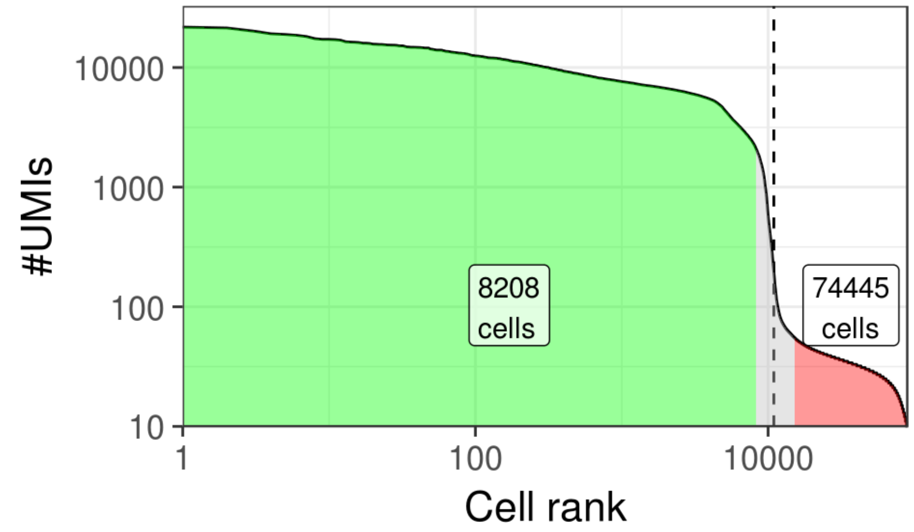

Cells quality

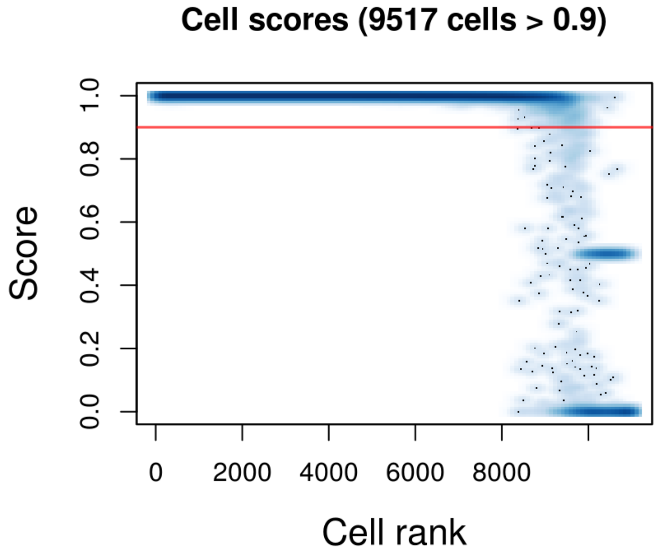

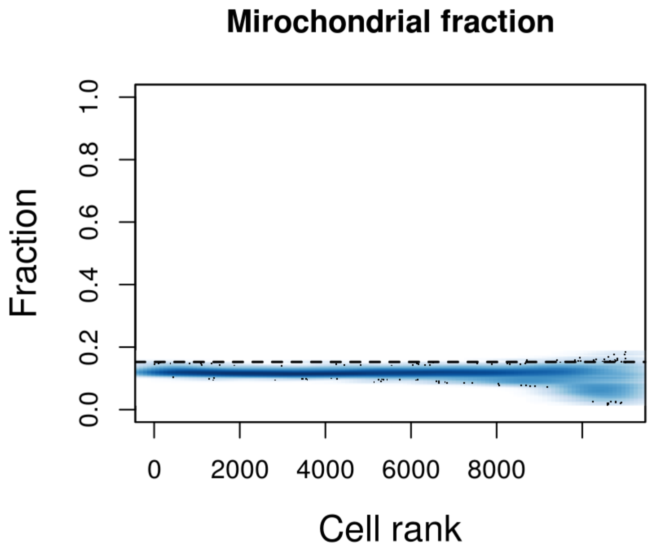

Saturation

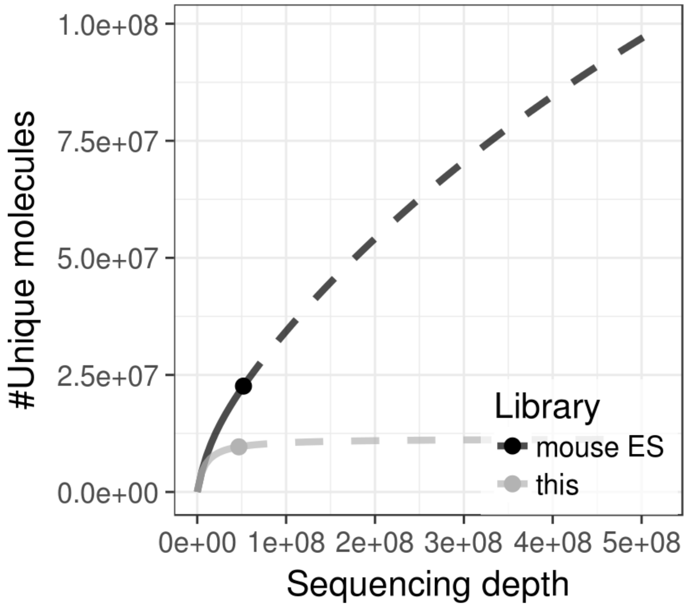

#UMIs per gene

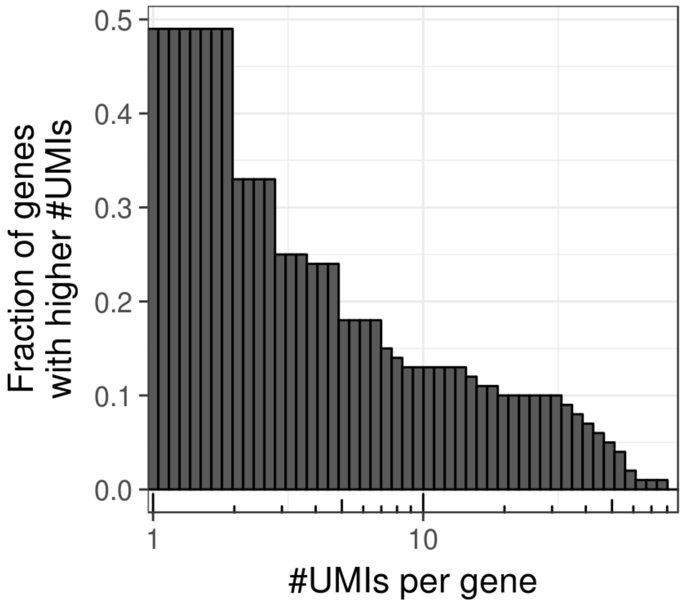

Statistics

Top genes:

Gene	#Molecules
ENSG00000251562	712828
ENSG00000140988	674780
ENSG00000147403	615551
ENSG00000229117	611132
ENSG00000156508	543493
ENSG00000167526	539853
ENSG00000142541	521459
ENSG00000231500	482001
ENSG00000186468	468263
ENSG00000137818	433870

Top UMIs:

UMI	#Molecules
CCCCG	142694
CCCGG	138656
CCGCA	127402
CCCGC	126550
ACCCG	120526
CCACA	117245
CACCA	116685
CCCAG	116533
CACCG	116252
CCCAC	112137
